# Designing highly stable ferrous selenide-black phosphorus nanosheets heteronanostructure via P-Se bond for MRI-guided photothermal therapy

**DOI:** 10.1186/s12951-021-00905-5

**Published:** 2021-07-06

**Authors:** Xuanru Deng, Hongxing Liu, Yuan Xu, Leung Chan, Jun Xie, Zushuang Xiong, Zheng Tang, Fang Yang, Tianfeng Chen

**Affiliations:** 1 College of Chemistry and Materials Science, Guangdong Provincial Key Laboratory of Functional Supramolecular Coordination Materials and Applications, Jinan University, Guangzhou, 510632 China; 2 Medical Imaging Center, The First Affiliated Hospital, Jinan University, Guangzhou, 510632 China; 3 Department of Urology, Guangzhou Institute of Urology, Guangdong Key Laboratory of Urology, the First Affiliated Hospital of Guangzhou Medical University, Guangzhou Medical University, Guangzhou, 510230 China

**Keywords:** Black phosphorus, P-se bond, Heteronanostructure, MRI, Photothermal therapy

## Abstract

**Background:**

The design of stable and biocompatible black phosphorus-based theranostic agents with high photothermal conversion efficiency and clear mechanism to realize MRI-guided precision photothermal therapy (PTT) is imminent.

**Results:**

Herein, black phosphorus nanosheets (BPs) covalently with mono-dispersed and superparamagnetic ferrous selenide (FeSe_2_) to construct heteronanostructure nanoparticles modified with methoxy poly (Ethylene Glycol) (mPEG-NH_2_) to obtain good water solubility for MRI-guided photothermal tumor therapy is successfully designed. The mechanism reveals that the enhanced photothermal conversion achieved by BPs-FeSe_2_-PEG heteronanostructure is attributed to the effective separation of photoinduced carriers. Besides, through the formation of the P-Se bond, the oxidation degree of FeSe_2_ is weakened. The lone pair electrons on the surface of BPs are occupied, which reduces the exposure of lone pair electrons in air, leading to excellent stability of BPs-FeSe_2_-PEG. Furthermore, the BPs-FeSe_2_-PEG heteronanostructure could realize enhanced T_2_-weighted imaging due to the aggregation of FeSe_2_ on BPs and the formation of hydrogen bonds, thus providing accurate PTT guidance and generating hyperthermia to inhabit tumor growth under NIR laser with negligible toxicity in vivo.

**Conclusions:**

Collectively, this work offers an opportunity for fabricating BPs-based heteronanostructure nanomaterials that could simultaneously enhance photothermal conversion efficiency and photostability to realize MRI-guided cancer therapy.

**Graphic abstract:**

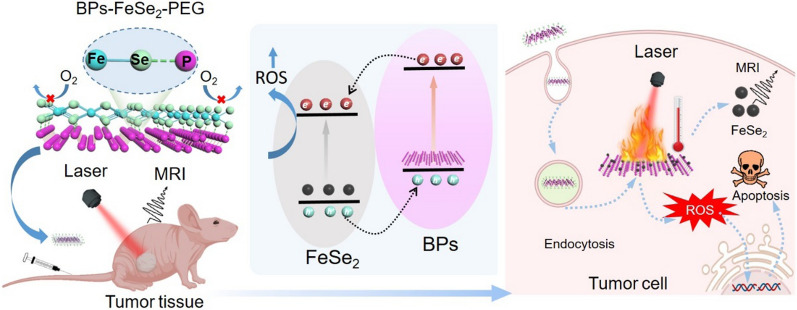

**Supplementary Information:**

The online version contains supplementary material available at 10.1186/s12951-021-00905-5.

## Background

The incidence of malignant tumors is increasing, which is one of the major diseases leading to human death [1]. Great efforts have been devoted to combating cancer, and traditional treatments such as chemotherapy, radiotherapy, and surgical resection have been developed [2]. However, therapeutic effects are not always satisfactory because of the metastasis and invasiveness of cancer. PTT relies on photothermal reagents to absorb near-infrared light to convert high temperature to achieve tumor ablation. Owing to the noninvasive, slight side effect and high temporal specificity, in recent years, PTT has attracted extensive attention. Therefore, it is necessary to find an efficient photothermal reagent to improve the effect of photothermal therapy. Generally, PTT reagents are divided into organic photothermal nanomaterials and inorganic nanomaterials [3–8]. Organic nanomaterials such as indocyanine green (ICG) [9], conjugated polymers [10] and dopamine melanin [11], have high biocompatibility and low toxicity, but poor photobleaching and thermal stability hinder their application in biomedicine. In contrast, inorganic nanomaterials such as graphene oxide [12], precious metal nanomaterials [13], transition metal carbides [14] and carbonitrides [15, 16] have been widely researched due to their easy modification, adjustable morphology and high physiological stability [17, 18]. However, the safety and biocompatibility of inorganic nanomaterials are still great challenges. Therefore, the design and search for photothermal reagents with safety, good stability, high photothermal conversion and good biocompatibility remain significant challenges.

Among the photothermal reagents, BPs, as emerging two-dimensional materials [19, 20], have unique properties such as huge surface area [21], high photothermal conversion efficiency [22], good biocompatibility [23] and eventual degradation to non-toxic phosphate or phosphonate, which have attracted increasing attention from researchers. Thus, BPs have been widely studied in antibacterial [24, 25], wound healing [26], antitumor [27–31] and other diseases [32, 33]. However, the lone pair electrons on the surface of BPs lead to high reactivity in air and water [34], which hindered the application of BP-based nanomaterials in the biomedical area. Fortunately, researchers have developed a wide range of strategies to functionalize the BPs surface to improve the stability, including the formation of solvent shells in the stripping process [35], surface coverage of two-dimensional materials[36], edge-selective functionalization [37] and oxide layer passivation [38]. Although these methods have achieved good results in improving the stability of black phosphorus, their biomedical applications are limited due to the complexity of preparation methods and unclear functional mechanism. Besides, designing therapeutic nanoparticles with MRI ability is significant for accurate cancer treatment. MRI provides a wealth of tumor information for pre-treatment diagnosis and a basis for real-time monitoring therapeutic progression to judge the curative effect. Some researchers have recently combined black phosphorus with MRI contrast reagents to build an integrated platform for theranostics. BPs were coated with tannic acid to chelate with Mn^2+^ ions, endowing the theranostic nanoplatform with T_1_ MRI-guided PTT ability [32, 39]. In other studies, BPs were loaded with Ce6 [40], Fe_3_O_4_ [41], MnO_2_ [42] and upconversion nanoparticles [43] to achieve imaging-guided PTT and PDT. Although black phosphorus nanoplatforms have been endowed with imaging ability [44] and improved photothermal conversion efficiency by the above strategies, the mechanism of enhanced photothermal conversion efficiency or degradability of nanomaterial was rarely clarified. Therefore, it is necessary to design BP-based nanoparticles with clear mechanisms, good degradability and MRI ability to combat cancer.

Fe and Se belong to biofriendly elements [45]. Fe-containing nanoparticles have been widely studied in MRI [46–48]. FeSe_2_, as a crucial class of transition metal dichalcogenide, has attracted intensive interest because of its excellent magnetic properties [49], good electrical conductivity [50] and high absorbance in the near-infrared (NIR) region [51]. However, FeSe_2_ contains Fe^2+^, which is easily oxidized to Fe^3+^ in vivo and in vitro [52]. We hypothesized that the lone pair electrons on the surface of black phosphorus could covalently combine with FeSe_2_ to improve each other’s stability. Besides, our previous studies have shown that BPs heteronanostructure materials could improve radiosensitization efficiency by increasing the chance of energy transfer [53, 54]. Therefore, we speculate that BPs are covalently bonded with FeSe_2_ to form heteronanostructure, which could improve the photothermal conversion efficiency and stability while facilitating T_2_ imaging.

**Scheme 1:**
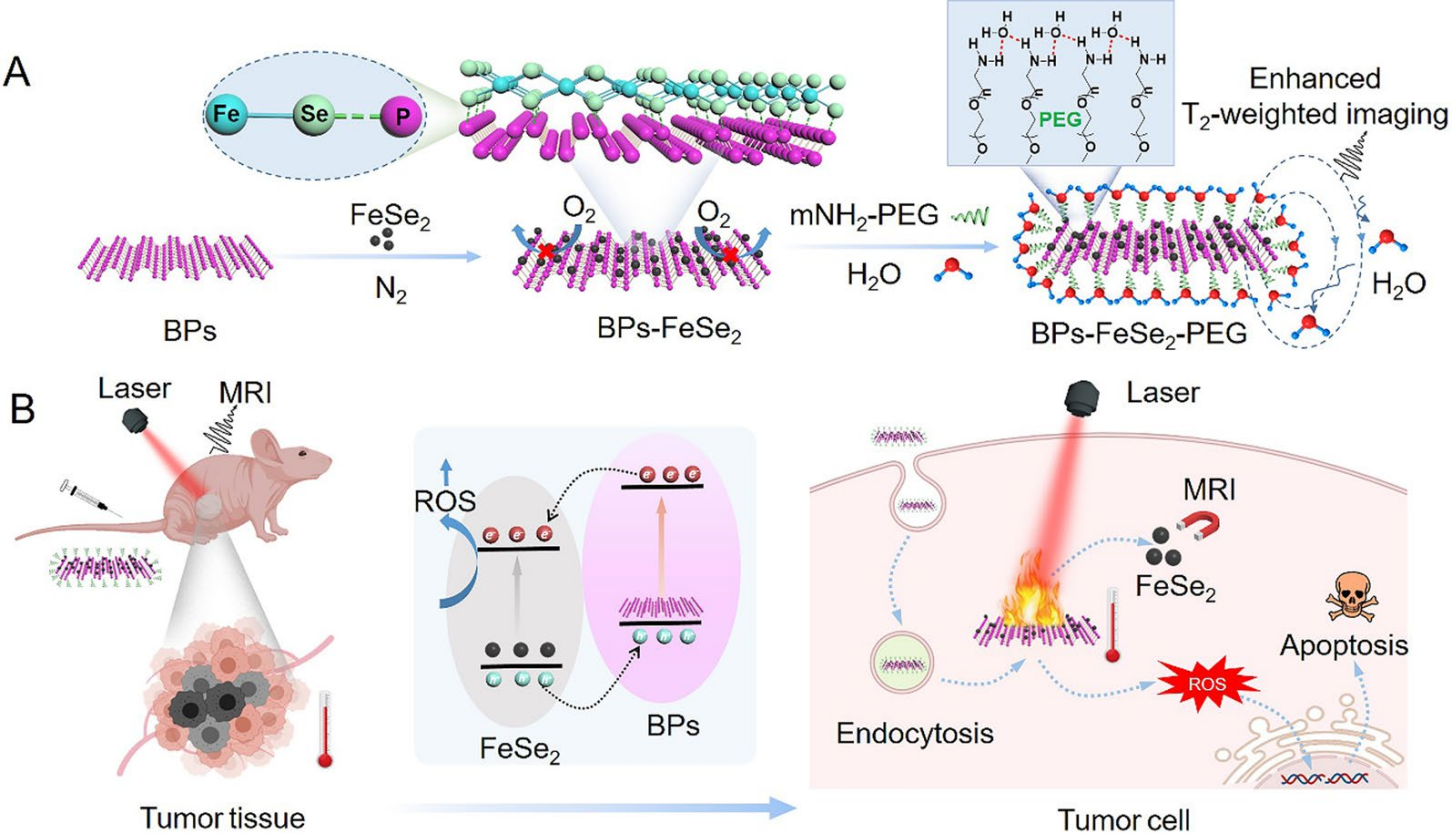
The schematic illustration of BPs-FeSe_2_-PEG heteronanostructure as MRI-guided agent for PTT. **A** the synthesis of BPs-FeSe_2_-PEG heteronanostructure. **B** the application and mechanism of BPs-FeSe_2_-PEG heteronanostructure as MRI-guided PTT agent

Herein, we designed and synthesized P-Se bonded BPs-FeSe_2_ heteronanostructure imaging therapeutic system as an efficient and stable photothermal reagent and MRI reagent to achieve T_2_-weighted imaging-guided PTT in Scheme 1. The benefit of the therapeutic system is the integration of the treatment function with MRI for precise treatment. Superior to previous studies, the as-prepared BPs-FeSe_2_-PEG heterostructures displayed higher photothermal conversion than free FeSe_2_-PEG or BPs-PEG upon NIR laser irradiation because the heterostructure enhanced the effective separation of photoinduced electrons and holes and reduced the recombination rate of photoinduced carriers. Photoinduced electrons and holes could be converted to hot carriers under irradiation, and then generate phonons to release the excess energy to return to equilibrium state via non-radiative recombination at the interface of FeSe_2_ and BPs. The excess energy caused enhanced photothermal performance of BPs-FeSe_2_ than that of single FeSe_2_ or BPs. Moreover, BPs-FeSe_2_-PEG heterostructure with MRI ability was acquired because of the superparamagnetism ability of FeSe_2_. Therefore, rational design of BPs-FeSe_2_-PEG heterostructures could simultaneously improve the deficiency of BPs and achieve the following advantages: (i) Photothermal stability of BPs-FeSe_2_-PEG is improved because of the formation of P-Se covalent bond. The oxidation degree of FeSe_2_ is weakened and the lone pair electrons on the surface of black phosphorus are occupied, which reduces the exposure of lone pair electrons in air to prevent the oxidation, leading to the excellent stability of BPs-FeSe_2_-PEG; (ii) Mechanism research shows that the BPs-FeSe_2_-PEG heteronanostructure presents enhanced photothermal conversion efficiency (η = 26.7%). In addition, ROS generation ability was also improved. These results were ascribed to the enhanced separation of photoinduced electrons and holes and the reduced recombination rate of photoinduced carriers during the formation of heterostructure; (iii) BPs-FeSe_2_-PEG could act as T_2_ MRI contrast agent to realize MRI-guided precision photothermal treatment of cancer. Besides, the BPs-FeSe_2_-PEG heteronanostructure could realize enhanced T_2_-weighted imaging due to the aggregation of FeSe_2_ on the surface of BPs and the formation of hydrogen bonds; (iv) Owing to the excellent thermal ablation effect, the therapeutic strategy of BPs-FeSe_2_-PEG combined with NIR irradiation shows a powerful antitumor ability; and (v) The nanomedicine has the characteristics of good degradability and no observable toxicity to major organs. Taken together, BPs-FeSe_2_-PEG heterostructures have great clinical potential for MRI-guided precision photothermal therapy.

## Materials and methods

### Materials

Bulk BP, N-Methyl pyrrolidone (NMP), ethanol, 1-ethyl-3-[3-(dimethylamino)-propyl] carbodiimide hydrochloride (EDC) and N-hydroxysuccinimide (NHS), cyclohexane, thiazolyl blue tetrazolium bromide (MTT), propidium iodide (PI) were obtained from Sigma-Aldrich. Oleyl amine, 1-Octadecene, Methoxy Polyethylene Glycol Amine and Poly (acrylic acid) (PAA) (M.W ~ 2000) were obtained from Macklin. FeCl_2_4H_2_O was obtained from Aladdin.

### Preparation BPs

Bulk BP (50 mg) was mixed with NMP solution (200 mL) and ultrasonic (960 W) for different times (8, 10 and 12 h) to obtain BPs with different thicknesses. The solution was then centrifuged (5000 rpm, 10 min) to remove the bulk BP crystals. The supernatant was centrifuged (8000 rpm, 10 min) to separate thick black phosphorus slices. Finally, the obtained supernatant was centrifuged (12,000 rpm, 60 min). The precipitate was washed with ethanol two times and stored in ethanol (20 mL) at 4 °C.

### Preparation of FeSe_2_

FeSe_2_ were synthesized according to previously reported method [55]. Under the N_2_ conditions, oleylamine (15 mL) and 1-octadecene (10 mL) were mixed in a three-necked flask and then maintained at 120 °C for 30 min. FeCl_2_4H_2_O (1 mmol) was rapidly added to the solution and kept at 120 °C. Simultaneously, the solution was intensely stirred for 30 min. Selenium powder (2 mmol) and oleylamine (4 mL) were mixed and heated under the condition of N_2_ until dissolved. The dissolved selenium powder was slowly injected into the flask. The temperature was rapidly raised to 150 °C in 10 min and maintained for 30 min. The reaction was cooled to room temperature under the N_2_ condition. The excess cyclohexane was added to the solution. Then the solution was centrifuged to obtain precipitate. Finally, the precipitate was dissolved in absolute ethyl alcohol and stored under the condition of N_2_.

### Preparation of FeSe_2_-PEG

FeSe_2_ ethanol solution was slowly added to the excess PAA solution under ultrasound. The excess ethanol and PAA were removed by centrifugation (8000 rpm, 5 min). Subsequently, the excess mPEG-NH_2_ solution was added to the PAA-FeSe_2_ aqueous solution. Then 5 mg EDCNHS was added to the FeSe_2_-PEG solution, stirred overnight at room temperature. FeSe_2_-PEG was collected by centrifugation (8000 rpm, 15 min). The obtained FeSe_2_-PEG was suspended in water.

### Preparation of BPs-PEG

10 mg mPEG-NH_2_ was dispersed in 10 mL 200 μg/mL BPs aqueous solution under ultrasound. The resulting BPs-PEG were centrifugated at 4000 rpm for 30 min and washed twice. The obtained BPs-PEG was dissolved in ultrapure water.

### ***Preparation of BPs-FeSe***_***2***_***-PEG***

3 mL of 4 mg/mL FeSe_2_ ethanol solution was slowly added to 3 mL of 1 mg/mL BPs ethanol solution (FeSe_2_: BPs 4:1), stirred for 12 h in the dark and centrifuged to remove the supernatant (3000 rpm, 15 min). Subsequently, the excess mPEG-NH_2_ solution was added to the solution and stirred overnight. The solution was then centrifuged to remove the supernatant (3000 rpm, 20 min). The obtained BPs-FeSe_2_-PEG was dissolved in water.

### ***Preparation of various FeSe***_***2***_***: BPs ratios of BPs-FeSe***_***2***_***-PEG***

FeSe_2_ ethanol solution (1 mg/mL) was slowly added to 1 mg/mL of BPs ethanol solution with different ratio (FeSe_2_: BPs 5:1, 4:1, 2:1, 1.5:1 and 1:1), stirred for 12 h in the dark and centrifuged to remove the supernatant (3000 rpm, 15 min). Subsequently, the excess mPEG-NH_2_ solution was added to the solution and stirred overnight. The solution was then centrifuged to remove the supernatant (3000 rpm, 20 min). The obtained BPs-FeSe_2_-PEG was dissolved in water.

### ***Characterization of the BPs-FeSe***_***2***_***-PEG***

The morphologies of FeSe_2_, BPs and BPs-FeSe_2_ were determined by transmission electron microscopy (TEM), high-resolution TEM and atomic force microscope (AFM). The structures of FeSe_2_, BPs and BPs-FeSe_2_ were measured by X-ray photoelectron spectroscopy (XPS), X-ray powder diffraction (XRD), UV–vis-NIR spectrophotometry and Raman scattering. The elemental distributions of BPs-FeSe_2_ were characterized by energy-dispersive X-ray spectroscopy (EDS).

### Stability of nanoplatforms

The UV–vis absorbance of the FeSe_2_-PEG, BPs-PEG and BPs-FeSe_2_-PEG dispersed in ultrapure water (pH = 7) were monitored at 0, 1, 2, 3, 4 and 5 days.

### Photothermal effects of nanoplatforms

To evaluate the effects of PEGylated FeSe_2_, BPs and BPs-FeSe_2_ used as photothermal agents, first, an equivalent concentration of PEGylated FeSe_2_, BPs and BPs-FeSe_2_ (120 μg/mL) were illuminated with different laser (0.5, 1, 1.5, 2 W/cm^2^). Second, a range of concentrations of PEGylated FeSe_2_, BPs and BPs-FeSe_2_ solution (600 μL) including 15, 30, 60 and 120 μg/mL were irradiated by NIR laser at 1.5 W/cm^2^ for 10 min.

### The photothermal conversion efficiency of nanoplatforms

PEGylated FeSe_2_, BPs and BPs-FeSe_2_ (1 mL) were added to the ultraviolet quartz dish. The photothermal conversion efficiency (η) of the cooling stage as measured through the following formula:

$$\eta = \frac{{h~s\Delta T_{{max}} {-}Q_{s} }}{{I\left( {1 - 10^{{ - A_{{808}} }} } \right)}}$$

where (mW/m^2^°C) represents the heat transfer coefficient, ${\text{S}}$(m^2^) means the surface area of the ultraviolet quartz dish, $\Delta T_{{max}}$ refers to the difference between the equilibrium and the ambient temperature. $Q_{s}$ is equal to the heat absorbed from the light by the ultraviolet quartz vessel containing water. $I$ stands for the laser power density. A_808_ means the absorbance at 808 nm. s = m c/T, where m refers to the solution mass, c means the specific heat capacity of the solution, and T is the ratio of time to − Inθ in the cooling process. FeSe_2_-PEG, BPs-PEG and BPs-FeSe_2_-PEG were added into ultraviolet quartz dishes, respectively.

### Degradation of nanoplatforms

The FeSe_2_-PEG, BPs-PEG and BPs-FeSe_2_-PEG (1 mg/mL, 1 mL) were suspended in PBS solution at pH 5.3, pH 5.3 with 1 mg/mL lysozyme, pH 7.4 and EJ cells lysates in RIPA with or without laser. The morphology changes of nanoplatforms were recorded by TEM.

### ***Magnetic properties of BPs-FeSe***_***2***_***-PEG***

The magnetic properties of FeSe_2_-PEG and BPs-FeSe_2_-PEG solution of different concentrations (0, 0.00125, 0.0025, 0.005, 0.01 and 0.02 mM) were recorded T_2_-weighted images by 1.5 T clinical MRI system (Signa HDxt, Milwaukee, WI).

### Cell culture

The human urinary bladder carcinoma cells EJ cells were purchased from American Type Culture Collection (ATCC, Manassas, Virginia, USA). EJ cells were cultured in DMEM medium with fetal bovine serum (10%), penicillin (100 units/mL), and streptomycin (50 units/mL) in a humidified incubator at 37 °C with 5% CO_2_ atmosphere.

### Anticancer efficacy of nanoplatforms

A range of concentration (from 0 to 50 μg/mL) of PEGylated FeSe_2_, BPs and BPs-FeSe_2_ incubated with EJ cells (density of 2 × 10^4^/mL, 100 μL) in the 96-hole wells for 8 h. The laser groups cells were exposed to laser (1.5 W/cm^2^, 5 min) and incubated for another 64 h. MTT solution was added to the cells. Then the supernatant was extracted and added 150 μL DMSO to each well. The absorption was measured at 570 nm by a microplate reader.

### Calcein-AM and PI staining

EJ cells incubated with PEGylated FeSe_2_, BPs and BPs-FeSe_2_ (25 μg/mL) for 8 h. The cells were irradiated with NIR laser (1.5 W/cm^2^, 5 min). The supernatant was removed. The same volume of PBS was added. The AM and PI were used to detect living cells and dead cells, respectively. Finally, the living and dead cells were recorded by fluorescence microscopy.

### Cellular uptake

Coumarin 6-labeled PEGylated FeSe_2_, BPs and BPs-FeSe_2_ were prepared to quantify the uptake of nanomaterials in cells. EJ cells were seeded at 6-well plates and incubated for 24 h. The same concentration of PEGylated FeSe_2_, BPs and BPs-FeSe_2_ (25 μg/mL) were added to EJ cells and incubated for 0, 1, 2, 4, 8 and 12 h. Next, the cells were collected and measured by flow cytometry [56].

### ***Intracellular localization of BPs-FeSe***_***2***_***-PEG in EJ cells***

Lysosomes and nucleus were stained with lysotracker and DAPI, respectively. BPs-FeSe_2_-PEG was incubated with EJ cells for 0, 1, 2, 4, 8 and 12 h. The fluorescence was capture by a fluorescence microscope (EVOS FL Auto Imaging System, AMAFD1000).

### Flow cytometric analysis

EJ cells were incubated with the same concentration of PEGylated FeSe_2_, BPs and BPs-FeSe_2_ (25 μg/mL) for 8 h. The laser irradiation groups were exposed to laser (1.5 W/cm^2^, 5 min). All of the groups were incubated for 72 h in total and stained with PI. Finally, the cells were analyzed by a flow cytometer.

### Annexin V-FITC and PI staining

EJ cells were incubated with PEGylated FeSe_2_, BPs and BPs-FeSe_2_ (25 μg/mL) for 8 h. The laser groups were exposed to the laser (1.5 W/cm^2^, 5 min). All of the groups were incubated for 72 h in total. The cells were collected by centrifugation and resuspended in 300 μL binding buffer. Finally, the cells were stained with Annexin V-FITC and PI according to the kit method and analyzed by a flow cytometer.

### ***Intracellular hydrogen peroxide and ***^***1***^***O***_***2***_*** generation***

EJ cells were incubated with 100 μg/mL of PEGylated FeSe_2_, BPs and BPs-FeSe_2._ The cells with or without laser (1.5 W/cm^2^, 1 min) were treated with DCFH-DA or 1, 3-diphenylisobenzofuran (DPBF) at a final concentration of 10 μM and 20 μM, respectively. Intracellular hydrogen peroxide and ^1^O_2_ generation were detected as the fluorescence intensity.

### MRI of BPs-FeSe_2_-PEG in vivo

FeSe_2_-PEG and BPs-FeSe_2_-PEG were intravenously injected (10 mg/kg). The T_2_-weighted signal of FeSe_2_ in tumor regions was collected at different time points (0, 2, 6, 12, 24 h) by a 3.0 T MR scanner (Bruker Biospin Corporation, Billerica, MA, USA).

### Antitumor activity in vivo

The nude mice used in this study were purchased from Beijing Vital River Laboratory Animal Technology Co, Ltd. All animal experiments were conducted under the approval of the Animal Experimentation Ethics Committee of Jinan University. 1 × 10^7^ EJ cells were suspended in 100 μL DMEM hypodermic and injected into nude mice. When the tumor reached about 120–150 mm^3^, the mice were intravenously injected with FeSe_2_-PEG, BPs-PEG and FeSe_2_-BPs-PEG (10 mg/kg). After 2 h of administration, the tumors of laser groups were irradiated with 808 nm laser (1.5 W/cm^2^, 10 min). The control mice were injected with an equal volume of saline. The tumor volume and body weight of each mouse was recorded per 2 days. The main organs, blood and tumors were collected after 21 days.

### Photothermal imaging in vivo

When the tumor volume of the nude mice reaches 150 mm^3^, the nude mice were randomly divided into four groups. PEGylated FeSe_2_, BPs and BPs-FeSe_2_ (10 mg/kg) are injected via intravenous. Then the temperature and images of the tumor area under laser irradiation (1.5 W/cm^2^, 10 min) were recorded by the infrared imager.

### Evaluation of antitumor effects by MRI

The nude mice of all groups were recorded the MRI images of tumor regions at 21 days of treatments by a 9.4 T MRI scanner. The necrotic degree of tumor issues was evaluated by the indexes of standard ADC, fast ADC and slow ADC.

### ***Pharmacokinetic study of BPs-FeSe***_***2***_***-PEG***

Three female SD mice (180–200 g) were intravenously injected BPs-FeSe_2_-PEG (4 mg/kg). Then we collected the blood from the eyes at 0.5, 1, 2, 4, 8, 12, 24, 48 and 72 h, respectively. We obtained the serum by centrifugation. Then the serum was digested with chloroazotic acid. The content of Se was determined by inductively coupled plasma mass spectrometry (ICP-MS).

### Statistical and synergy analysis

Statistical analysis was performed using the SPSS statistical program version 13 (SPSS Inc. Chicago, IL). All the experiments were carried out at least in triplicate. The results were expressed as means ± SD. Differences between the two groups were analyzed by the two-tailed Students *t*-test. Differences of *P* < *0.05 *(*)*, P* < *0.01 *(**)* or P* < *0.001 *(***) was indicated.

## Results and discussion

### Preparation and characterization of BPs-FeSe_2_

**Fig. 1:**
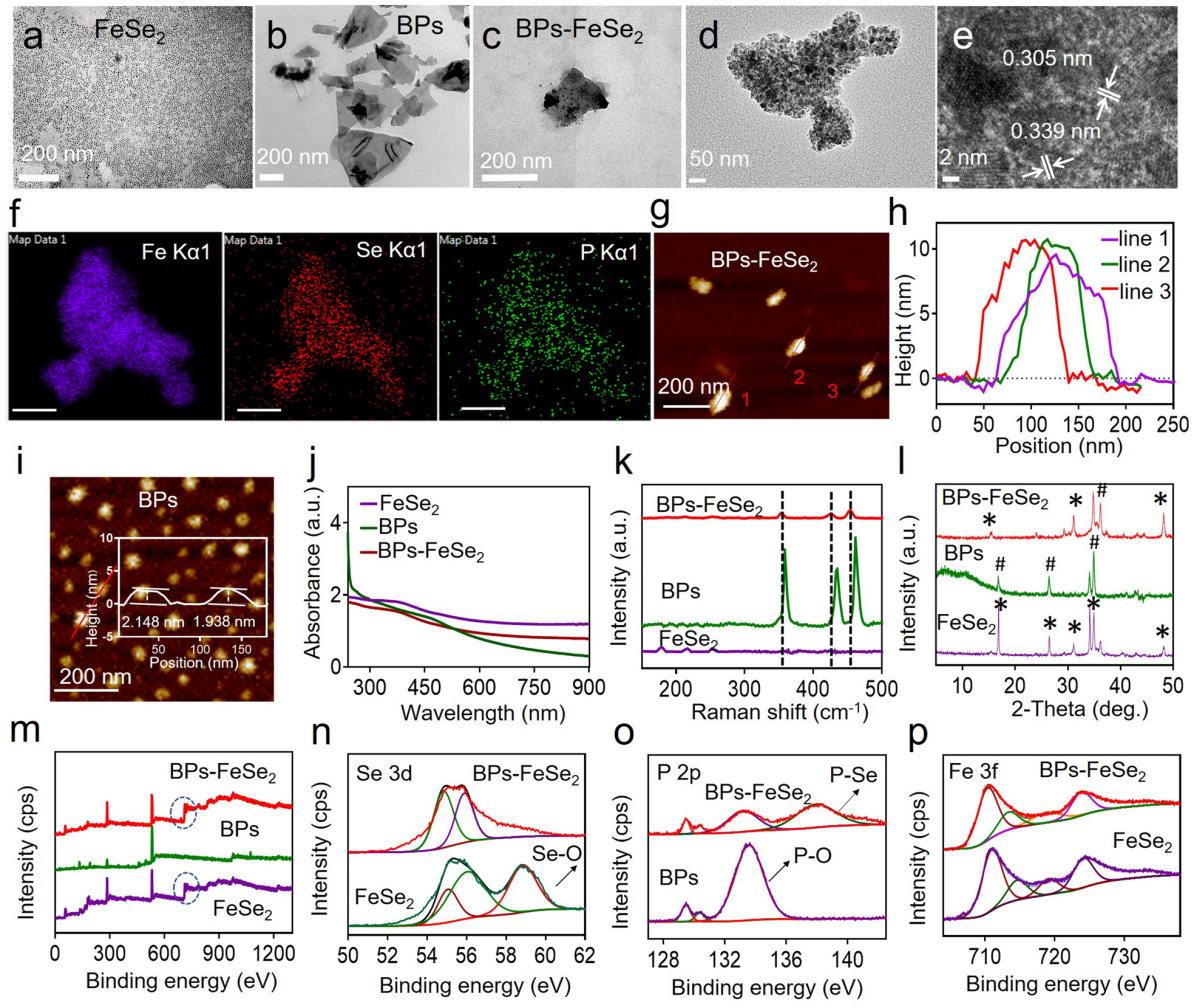
Characterization of BPs-FeSe_2_. TEM image of **a** FeSe_2_; **b** BPs; and **c** BPs-FeSe_2_. **d** HR-TEM images of BPs-FeSe_2_. **e** Lattice fringes of **d**. **f** EDS elemental mapping images of BPs-FeSe_2_ (scale bar = 50 nm). **g** AFM image of BPs-FeSe_2_. **h** Height profiles along the red lines in **g**. **i** AFM image of BPs. **j** UV–vis absorption spectra of FeSe_2_, BPs and BPs-FeSe_2_. **k** Raman spectra of FeSe_2_, BPs and BPs-FeSe_2_. **l** XRD spectra of FeSe_2_, BPs and BPs-FeSe_2_. **m**–**p** XPS spectra of P 2p, Se 3d and Fe 2p compared with FeSe_2_, BPs or BPs-FeSe_2_

This study designed and synthesized the BPs-FeSe_2_ nanosystem, which was featured with enhanced photothermal conversion efficiency and MRI-guided therapy. FeSe_2_ was covalent on the surface of BPs to enable MRI-guided therapy. The BPs could then achieve photothermal conversion. The FeSe_2_ nanoparticles were synthesized via the heat injection method. TEM was used to observe the morphology of BPs-FeSe_2_. The uniform size of FeSe_2_ in Fig. 1a is about 8 nm with good dispersion. The BPs with a size of about 200 nm were obtained through the classical liquid-phase exfoliation method [57] shown in Fig. 1b. The BPs dissolved in ethanol were injected into as-synthesized FeSe_2_ nanoparticles and reacted overnight under N_2_ atmosphere at room temperature. As shown in Fig. 1c, FeSe_2_ nanoparticles were uniformly dispersed on BPs. Interestingly, we found that the thickness of BPs would affect the density of FeSe_2_ decorated on the surface of BPs. BPs with various thicknesses were obtained under different ultrasound time. By adding BPs with a thickness of about 2.4 nm, the FeSe_2_ nanoparticles were completely decorated on the surface (Additional file 1: Figure S1e, f). As the thickness of BPs increased, the density of FeSe_2_ nanoparticles decorated on the BPs was reduced (Additional file 1: Figure S1a–d). We speculated that the thinner the BPs, the more lone pair electrons are exposed, leading to the easier covalent binding to FeSe_2_. Meanwhile, by changing the amount of added FeSe_2_, the density of FeSe_2_ modified on the surface of BPs could also be adjusted (Additional file 1: Figure S2). When the FeSe_2_: BPs ratio was 1:1, only a small part of FeSe_2_ was modified on the surface of BPs. With the increase of FeSe_2_ addition, the density of FeSe_2_ nanoparticles on the BPs increased. Notably, when FeSe_2_ was added at the FeSe_2_: BPs ratio of 4:1, FeSe_2_ nanoparticles were completely modified on the surface of BPs. However, if an excess of FeSe_2_ was added (at the FeSe_2_: BPs ratio of 5:1), a small part of the FeSe_2_ was scattered around the BPs. Finally, we chose the thickness of BPs about 2 nm and the feeding FeSe_2_: BPs ratio of 4:1 to prepare BPs-FeSe_2_ for subsequent experiments. The interplanar spacing of 0.339 nm and 0.305 nm of high-resolution TEM (HR-TEM) in Fig. 1d, e; were matched with the (0 2 1) plane of P and the (1 1 1) plane of FeSe_2_, respectively. The distribution of Fe, Se and P elements in the BPs-FeSe_2_ nanosheet was observed in EDS data, which confirmed that BPs-FeSe_2_ were successfully established (Fig. 1f). As shown in Fig. 1g, h, the atomic force microscopy (AFM) image revealed the morphology of the BPs-FeSe_2_, and the height measured was about 10 nm, where the thickness of BPs was about 2 nm (Fig. 1i) and the height of FeSe_2_ was about 8 nm. As shown in Fig. 1j, compared with the absorption spectra of FeSe_2_, the significant representative absorption profiles in the NIR region are fully retained in BPs-FeSe_2_ due to the intrinsic absorption characteristics of BPs, providing rationale for photothermal mechanism. The UV–vis absorption spectra in Additional file 1: Figure S3 exbibited that BPs-FeSe_2_ showed strong optical absorption from NIR-I to NIR-II window, suggesting that the BPs-FeSe_2_ heteronanostructure was a potential candidate for application in NIR-II PTA. Raman scattering was further introduced to characterize the FeSe_2_, BPs and BPs-FeSe_2_ as in Fig. 1k. BPs-FeSe_2_ showed three representative Raman peaks at 358.4, 434.8 and 433.1 cm^−1^ ascribed to A_g_^1^, B_2g_ and A_g_^2^ of BPs, respectively, confirming the introduction of FeSe_2_ on the BPs. The structure of the nanoparticles was studied by X-ray diffraction (XRD). As shown in Fig. 1l, the representative diffraction peaks of BPs-FeSe_2_ were matched with FeSe_2_ and BPs, revealing the co-existence of FeSe_2_ and BPs. X-ray photoelectron spectroscopy (XPS) was used to detect the chemical composition and binding energies of FeSe_2_, BPs and BPs-FeSe_2_, respectively. As shown in Fig. 1m, full-spectrum comparison confirmed the existence of FeSe_2_ on BPs. It should be noted that there is a peak of 58.91 eV in the Se 3d high-energy region of FeSe_2_, referring to Se-O, indicating that the individual FeSe_2_ is oxidized, while the absence of analogous peaks in the BPs-FeSe_2_ Se 3d spectrum indicates that the oxidation of FeSe_2_ in BPs-FeSe_2_ is negligible in Fig. 1n. As shown in Fig. 1o, the peak of P 2p at 133.9 eV was attributed to P-O, which indicated that both BPs and BPs-FeSe_2_ were oxidized, but the oxidation degree of BPs-FeSe_2_ was remarkably weaker than that of bare BPs. Besides, the characteristic peak of BPs-FeSe_2_ in 138.25 eV represented the P-Se bond, while P alone did not appear. As shown in Fig. 1p, the two strong peaks at 724.68 and 710.87 eV are associated with Fe 2p_1/2_ and Fe 2p_3/2_, confirming the formation of FeSe_2_. These results revealed that the covalent P-Se bond between FeSe_2_ and BPs could reduce the oxidation of BPs and FeSe_2_. According to previous reports, BPs are sensitive to water and oxygen, and FeSe_2_ contains Fe^2+^, so they are easily oxidized. Owing to the covalent P-Se bond, the oxidation degree of FeSe_2_ is weakened, and the lone pair electrons on the surface of black phosphorus are occupied, which reduces the exposure of lone pair electrons in air to prevent the oxidation, leading to excellent stability of BPs-FeSe_2_-PEG. Taken together, the BPs-FeSe_2_ was successfully synthesized based on the above results.

### ***Stability and photothermal performance of BPs-FeSe***_***2***_***-PEG***

**Fig. 2:**
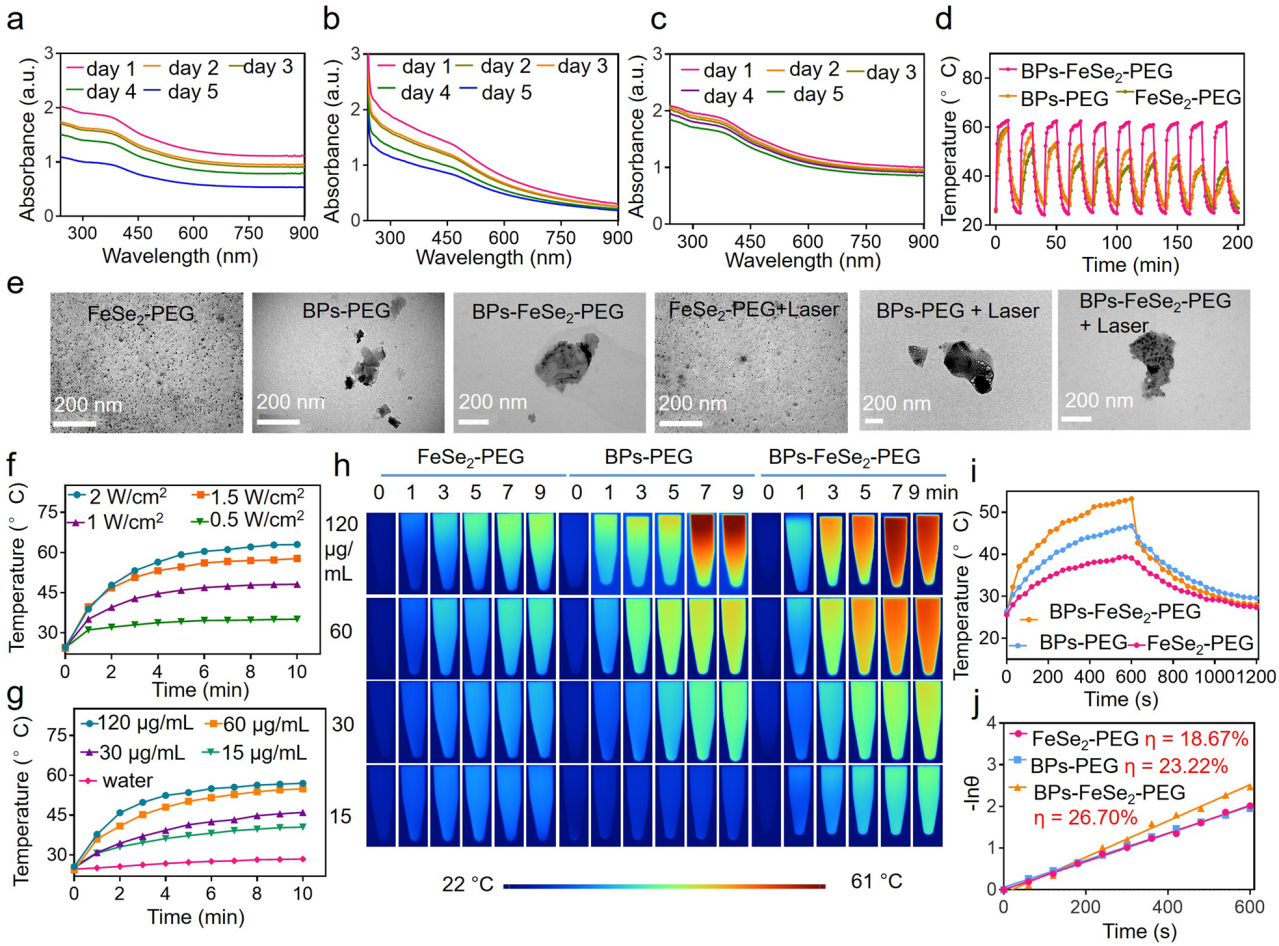
Stability of BPs-FeSe_2_-PEG. **a**–**c** UV–vis absorption spectra of PEGylated FeSe_2_, BPs and BPs-FeSe_2_ solutions for 5 days. **d** Photostability of PEGylated FeSe_2_, BPs and BPs-FeSe_2_ under NIR laser irradiation for 10 cycles (120 μg/mL, 1.5 W/cm^2^, 10 min). **e** TEM images of PEGylated FeSe_2_, BPs and BPs-FeSe_2_ after 10 cyclic irradiation. **f** Temperature curves of BPs-FeSe_2_-PEG solution (120 μg/mL, 10 min) under the irradiation of NIR laser various power (0.5, 1, 1.5, 2 W/cm^2^). **g** Temperature curves of BPs-FeSe_2_-PEG solution at different concentration (0, 15, 30, 60, 120 μg/mL) with NIR laser irradiation (1.5 W/cm^2^, 10 min). **h** Infrared thermal image. **i** The temperature increment curve and the cooling curve of PEGylated FeSe_2_, BPs and BPs-FeSe_2_. **j** Line curve of − Inθ vs time obtained from the cooling period of **i**

To further improve the biomedical application of the as-synthesized BPs-FeSe_2_, mNH_2_-PEG was used to coat it to enhance its water solubility and bioavailability (Additional file 1: Figure S4). Then the water-soluble PEGylated FeSe_2_, BPs and BPs-FeSe_2_ were obtained. To evaluate the influence of FeSe_2_ on the stability of BPs, the PEGylated FeSe_2_, BPs and BPs-FeSe_2_ were dispersed in ultrapure water for 5 days. The corresponding absorption spectra were shown in Fig. 2a–c. In the ultraviolet and near-infrared regions, PEGylated FeSe_2_, BPs and BPs-FeSe_2_ showed typical broad absorption bands. Nevertheless, the absorbance intensity of BPs-FeSe_2_-PEG only showed a negligible change, while that of FeSe_2_-PEG and BPs-PEG decreased remarkably with time. Interestingly, BPs-FeSe_2_-PEG still showed good stability on the 14th day (Additional file 1: Figure S5), while the BPs alone was almost completely degraded. The decrease in the absorbance of BPs may be attributed to the existence of lone pair electrons on the surface. As a result, when BPs were exposed to air, they were easily oxidized to PxOy. Besides, the phosphoric acid was formed when BPs were exposed to aqueous media. Both reasons eventually lead to the degradation of BPs. Photothermal stability is one of the significant factors to be envisioned as photothermal reagents. Consequently, we investigated the photothermal stability of PEGylated FeSe_2_, BPs and BPs-FeSe_2_ in water under NIR laser irradiation. PEGylated FeSe_2_, BPs and BPs-FeSe_2_ aqueous solutions were exposed to NIR laser to monitor the temperature change (laser on). Then, the solutions were cooled to room temperature (laser off). Interestingly, the photothermal effect of BPs-FeSe_2_-PEG showed no noticeable decrease during 10 cycles under irradiation for 200 min. By contrast, the photothermal effect of FeSe_2_-PEG and BPs-PEG decreased significantly in the same condition (Fig. 2d), which indicated the excellent thermal stability of BPs-FeSe_2_-PEG. Besides, the TEM images in Fig. 2e revealed the morphology of BPs-FeSe_2_-PEG, a negligible change after irradiation, indicating its high photostability. Then the stability of BPs-FeSe_2_-PEG in different simulating intracellular environments was investigated, and the morphological changes were observed. As shown in Additional file 1: Figure S6, the morphology was obviously dissociated after treatment with pH at 5.3 and lysozyme, while BPs-FeSe_2_-PEG in pH at 7.4 and 6.8 showed no significant change. Moreover, the BPs-FeSe_2_-PEG markedly dissociated in the condition of EJ bladder cell lysate. The above results collectively revealed that BPs-FeSe_2_-PEG possessed excellent water stability in simulated normal biological systems and degradability in simulated tumor systems, which endowed BPs-FeSe_2_-PEG great potential as a photothermal agent. Considering the great potential as a photothermal agent, the photothermal performance of BPs-FeSe_2_-PEG was investigated by exposing various concentrations of BPs-FeSe_2_-PEG to NIR laser. The temperature change was recorded at intervals using an IR thermal driver. As shown in Fig. 2f–h and Additional file 1: Figure S7a–d, those photothermal curves disclosed concentration and power-dependent heating effects and reached equilibrium within 5 min. When the BPs-FeSe_2_-PEG solution was exposed to laser at a density of 1.5 W/cm^2^ for 10 min, the temperature swiftly rose to 57 °C, which could ablate the tumor. Nevertheless, only 4 °C of increased temperature was detected in water, indicating the good photothermal effect of BPs-FeSe_2_-PEG. Besides, the photothermal conversion efficiency (η) of PEGylated FeSe_2_, BPs and BPs-FeSe_2_ were calculated referring to previous methods (Fig. 2i, j). The value of BPs-FeSe_2_-PEG was 26.7%, which was higher than that of FeSe_2_-PEG (18.67%), BPs-PEG (23.22%) and other classical inorganic photothermal reagent [58–60].

### Photothermal mechanism of BPs-FeSe_2_ heteronanostructure

**Fig. 3:**
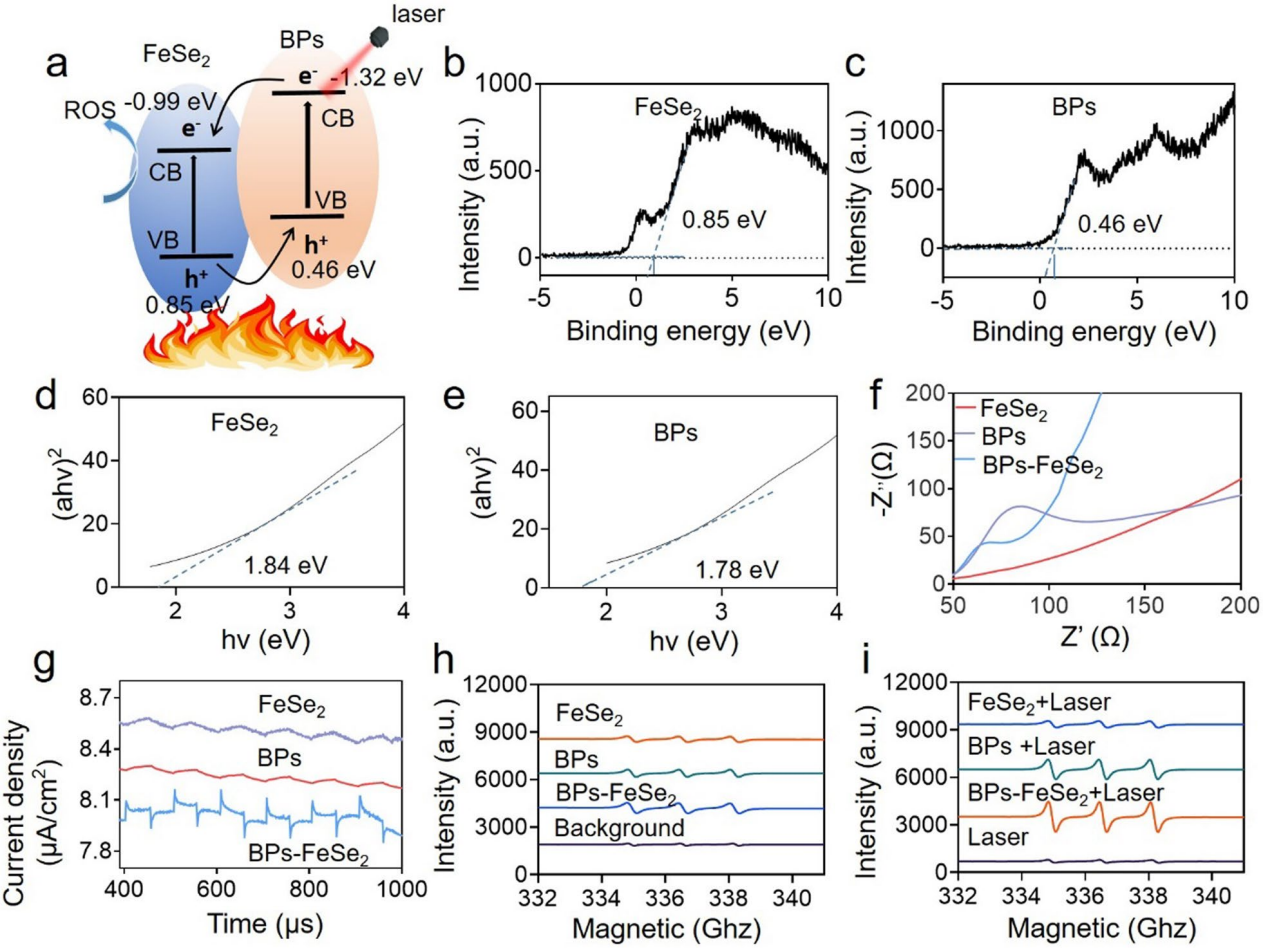
Photothermal mechanism of BPs-FeSe_2_. **a** Photothermal mechanism of the charge transfer of BPs-FeSe_2_ under light irradiation. **b**, **c** The valance band of FeSe_2_ and BPs. **d**, **e** The bandgap of FeSe_2_ and BPs is calculated by KubelkaMunk equation. **f** EIS spectra of FeSe_2_, BPs and BPs-FeSe_2_. **g** Photocurrent with FeSe_2_ and BPs responded to the on/off light irradiation. **h**, **i** ESR spectra of FeSe_2_, BPs and BPs-FeSe_2_ without/with NIR laser for ^1^O_2_ (100 μg/mL, 1.5 W/cm^2^, 5 min)

To understand the mechanism of the enhanced photothermal performance of BPs-FeSe_2_ heteronanostructure, the physical properties of FeSe_2_ and BPs were investigated. As shown in Fig. 3b, c, the valance band (VB) values of FeSe_2_ and BPs were identified by XPS spectra and calculated to be about 0.85 eV and 0.46 eV, respectively. The band gaps (Eg) of FeSe_2_ and BPs obtained from UV–vis-NIR diffuse spectra were about 1.84 eV and 1.78 eV, respectively (Fig. 3d, e). Electrochemical impedance spectroscopy (EIS) revealed the smallest diameter of BPs-FeSe_2_, suggesting that BPs-FeSe_2_ heteronanostructure had the lowest impedance and electron–hole recombination rate (Fig. 3f). The photocurrent responses were detected under irradiation, which provided evidence of efficient separation and transmission of photoinduced charges. As shown in Fig. 3g, a photocurrent had a response when turning on the light, but no response was observed when the light was off, confirming the efficient charge separation and transmission. Moreover, BPs-FeSe_2_ had the highest photocurrent response, indicating the fast separation of electron–hole pairs and low rate of electron–hole pairs recombination between FeSe_2_ and BPs. To illustrate the transferring mechanism of electron–hole pairs, the types of active radicals generated by BPs-FeSe_2_ under irradiation were detected by electron spin resonance (ESR). As shown in Fig. 3h, i, BPs-FeSe_2_ under irradiation for 5 min showed the most strong ^1^O_2_ signal than other groups, which indicated the generation of ^1^O_2_ radical. The possible mechanism of FeSe_2_ heteronanostructures enhanced photothermal performance was proposed. The energy band and electron transfer were vividly illustrated in Fig. 3a. The valence electrons (VB) of FeSe_2_ and BPs were excited into the conduction band (CB) of FeSe_2_ and BPs after irradiation, which caused the generation of electron–hole pairs. The photoinduced electrons transferred from the CB of BPs to the CB of FeSe_2_, while the holes transferred from the VB of FeSe_2_ to the VB of BPs, indicating the effective separation of photoinduced electrons and holes. The low recombination rate means an enhanced lifetime of photogenerated carriers, leading to more photogenerated carriers generating ^1^O_2_. Besides, under irradiation, the photogenerated carriers could be changed into hot carriers and then produce phonons to release excess energy to return to equilibrium state via non-radiative recombination at the interface of FeSe_2_ and BPs. The excess energy caused enhanced photothermal performance of BPs-FeSe_2_ than that of single FeSe_2_ or BPs. In summary, the radiative recombination of the holes and electrons was effectively inhabited and the lifetime of the holes and electrons was increased in the BPs-FeSe_2_ heterostructure, which resulted in a high degree of photothermal conversion. A lot of researches have demonstrated that the heterostructure could enhance the effective separation of photoinduced electrons and holes, delay the recombination of electron–hole pairs, and increase the ROS yield during the electron–hole pair transfer under laser irradiation. For instance, Wu et al. proved that a heterojunction which was composed of a photoresponsive metal–organic framework and Prussian blue could exhibit an enhanced photothermal effect [61]. Hence, we infer that, in general, a heterostructure, which could enhance the effective separation of photoinduced electrons and holes and delay the recombination of electron–hole pairs under laser irradiation, can lead to the improvement of photothermal efficiency of this heterostructure.

### Anticancer efficacy of BPs-FeSe_2_-PEG in vitro

**Fig. 4:**
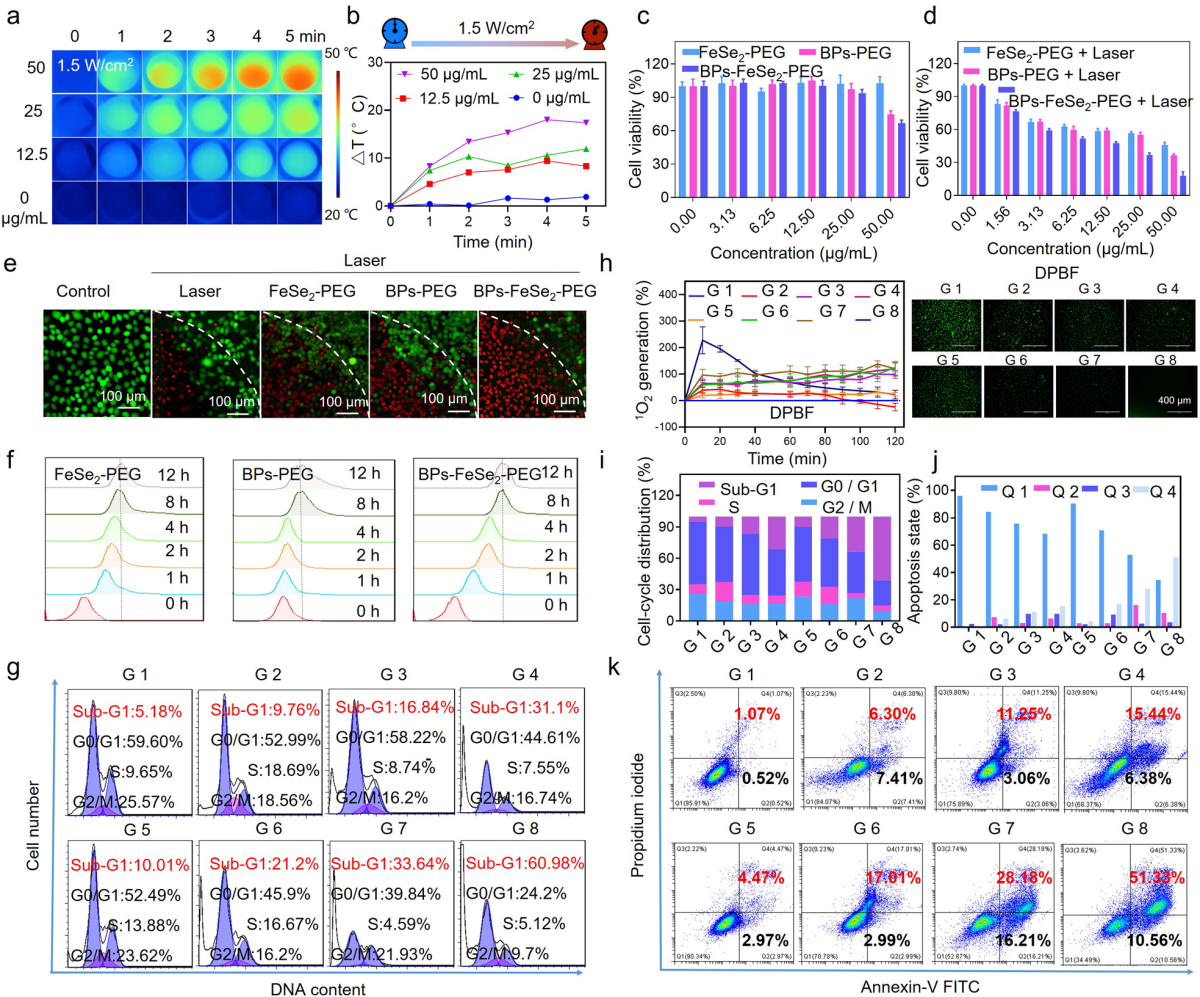
Anticancer efficacy of BPs-FeSe_2_-PEG in vitro. **a** Infrared thermal images and **b** temperature change of EJ cells incubated with different concentrations BPs-FeSe_2_-PEG under NIR laser (1.5 W/cm^2^, 5 min). **c**, **d** The EJ bladder cell viability of PEGylated FeSe_2_, BPs and BPs-FeSe_2_ at various concentrations with/without NIR laser for 72 h. (1.5 W/cm^2^, 5 min). **e** Fluorescence images of EJ cells stained with calcein-AM (green) and PI (red) under different treatment (25 μg/mL, 1.5 W/cm^2^, 5 min). **f** Cellular uptake analysis results of the same concentration of coumarin-6 labeled PEGylated FeSe_2_, BPs and BPs-FeSe_2_ in EJ cells by flow cytometry analysis (25 μg/mL). **g** Cell cycle arrest in EJ cells induced the same concentration of PEGylated FeSe_2_, BPs and BPs-FeSe_2_ with/without NIR laser (25 μg/mL, 1.5 W/cm^2^, 5 min). G 1: Control; G 2: FeSe_2_-PEG; G 3: BPs-PEG; G 4: BPs-FeSe_2_-PEG; G 5: laser; G 6: FeSe_2_-PEG + laser; G 7: BPs-PEG + laser; G 8: BPs-FeSe_2_-PEG + laser. **h** The generation of singlet oxygen during 120 min and representative fluorescence photos at 120 min in EJ bladder cells under different treatment (100 μg/mL, 1.5 W/cm^2^, 1 min). **i** Histogram of cell cycle phase of EJ bladder cells under different treatments matched with **g**. **j** Histogram of apoptosis of EJ bladder cells detected by AnnexinV/PI Staining matched with **k**. **k** Annexin V-FITC/PI double staining results under different treatment (25 μg/mL, 1.5 W/cm^2^, 5 min)

It is worth examining the temperature variation of BPs-FeSe_2_-PEG at the cellular level under NIR laser irradiation. Bladder cancer tumor cells (EJ cells) were used as models to analyze the effects of photothermal therapy on nanoparticles at the cellular level. As shown in Fig. 4a, b and Additional file 1: Figure S8a, b; the temperature rising rate was concentration-dependent and power-dependent. These results showed that at the same concentration (50 μg/mL), the temperature increased by 18 °C at a laser density of 1.5 W/cm^2^, which was similar to that of 2 W/cm^2^, but only 7.5 °C at the laser density of 1 W/cm^2^. Therefore, 1.5 W/cm^2^ was determined as a suitable laser density for subsequent experiments. Overall, the results revealed that BPs-FeSe_2_-PEG has excellent potential as a photothermal reagent. Then, in vitro phototherapeutic effects of BPs-FeSe_2_-PEG against EJ bladder cells were evaluated by 3-(4, 5-dimethylthiazol-2-yl)-2, 5-diphenyltetrazolium bromide (MTT) assay. As shown in Fig. 4c, d, after NIR laser irradiation, 63.16% of the BPs-FeSe_2_-PEG (25 μg/mL)  treated cells were killed, which was higher than those treated with FeSe_2_-PEG (41.64% cell death) and BPs-PEG (44.89% cell death). The therapeutic efficacy of BPs-FeSe_2_-PEG showed a dose-dependent manner.

Besides, the effect of the nanoparticle on SVHUC-1 cells was studied. As shown in Additional file 1: Figure S9, the BPs-FeSe_2_-PEG exhibited a high degree of safety to normal cells. To visually disclose the anticancer activities of PEGylated FeSe_2_, BPs and BPs-FeSe_2_ combined with PTT, calcein AM and PI co-staining was performed. As shown in Fig. 4e, in the area of laser irradiation illumination, the cell death was induced by PEGylated FeSe_2_, BPs and BPs-FeSe_2_. The clear dividing line could be observed between dead (red) and live (green) cells. The cells treated with BPs-FeSe_2_-PEG showed the most significant tendency to die, attributed to the great photothermal conversion efficiency, leading to the highest temperature among them. In contrast, cells only treated with NIR laser had slight cell death, revealing higher anticancer efficacy of BPs-FeSe_2_-PEG. Then we utilized flow cytometry to study the intracellular uptake of coumarin-6 labeled PEGylated FeSe_2_, BPs and BPs-FeSe_2_ in EJ bladder cells at different times. The cellular uptake of PEGylated FeSe_2_, BPs and BPs-FeSe_2_ was time-dependent and reached saturation within 8 h (Fig. 4f). Furthermore, to vividly observe the intracellular localization of coumarin-6-labeled BPs-FeSe_2_-PEG in EJ cells, the nucleus and lysosomes were stained using lysotracker (red) and DAPI (blue), respectively. As shown in Additional file 1: Figure S10, the cytoplasm of EJ cells obviously showed green fluorescence after 1 h. Moreover, the overlapping color of red and green fluorescence can be found after 8 h, which is consistent with cellular uptake. As a result, the BPs-FeSe_2_-PEG enter EJ bladder cells through endocytosis, which was time-dependent. To study the biological effects of BPs-FeSe_2_-PEG heteronanostructure combination with laser, flow cytometry was employed to investigate EJ bladder cells cycle distribution proportion. As shown in Fig. 4g, i, the sub-G 1 peak of laser alone group was 10.01%. However, it should be noted that the sub-G 1 peak of BPs-FeSe_2_-PEG combination with laser group remarkably enhanced to 60.98%. Those results provided evidence that BPs-FeSe_2_-PEG combined with laser-induced cell apoptosis inhibited the growth of EJ bladder cells. The flow cytometry studies illuminate that BPs-FeSe_2_-PEG exhibits more phototherapeutic effects than FeSe_2_-PEG and BPs-PEG, which could be ascribed to good stability and great photothermal conversion efficiency of BPs-FeSe_2_-PEG. To further illustrate the antitumor mechanisms of BPs-FeSe_2_-PEG, we used Annexin V-FITC / PI double labeling kit to analyze EJ cell apoptosis with/without laser irradiation. As shown in Fig. 4j, k, BPs-FeSe_2_-PEG mainly enhanced cell apoptosis in the late-stage. Reactive oxygen species (ROS) include superoxide anions (^−^O_2_), hydroxyl radical (OH), hydrogen peroxides (H_2_O_2_) and singlet oxygen (^1^O_2_). The overproduction of ROS leads to cell apoptosis [62]. The production of hydrogen peroxide and singlet oxygen induced by cotreatment was analyzed by 2, 7-dichlorodihydrofluorescein diacetate (DCFH-DA) and 1, 3-diphenylisobenzofuran (DPBF) probes, respectively. The combination treatments enhanced the level of H_2_O_2_ in EJ cells shown in Additional file 1: Figure S11. Besides, as shown in Fig. 4h, the intracellular ^1^O_2_ level induced by BPs-FeSe_2_-PEG combination with laser increased remarkably in 40 min than that of other treatment groups. The fluorescence images were consistent with the experimental results. The results indicated that the combination of laser and BPs-FeSe_2_-PEG elevated the generation of ^1^O_2_ and H_2_O_2_, which caused the tumor apoptosis.

### Imaging-guided therapy in vivo

**Fig. 5:**
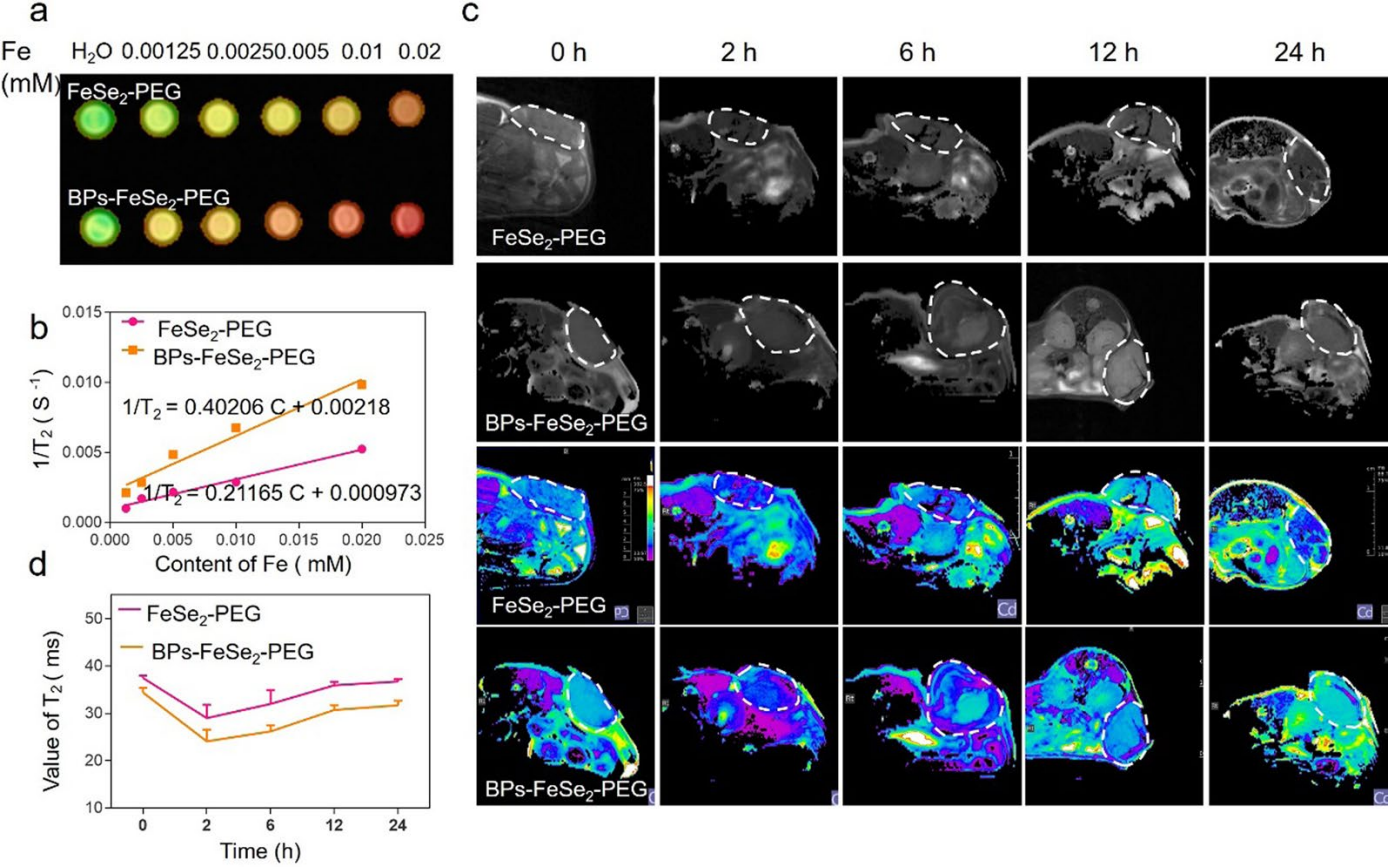
Imaging analysis of BPs-FeSe_2_-PEG. **a** T_2_-MRI images of FeSe_2_-PEG and BPs-FeSe_2_-PEG solution at different concentrations (0–0.02 mM) in vitro. **b** T_2_ relaxation rates of FeSe_2_-PEG and BPs-FeSe_2_-PEG solution under different concentrations. **c** T_2_-weighted MRI images of mice bearing EJ bladder tumor after intravenous injection of FeSe_2_-PEG and BPs-FeSe_2_-PEG (10 mg/kg) for 0, 2, 6, 12 and 24 h. **d** The intensity of MRI signals matched with **c**

MRI provides abundant tumor information for pre-treatment diagnosis. It provides a basis for real-time monitoring of therapeutic progression, judging the curative effect, and realizing the precise treatment of cancer. FeSe_2_ illustrated the superparamagnetic nature as previously reported. The FeSe_2_ core could react with H_2_O and needed to be coated with PEG to dissolve in aqueous solution. Then we evaluated different concentrations (Fe concentration 0, 0.00125, 0.0025, 0.005, 0.01, 0.02 mM) of FeSe_2_-PEG and BPs-FeSe_2_-PEG applied for MRI analysis in vitro. T_2_-weighted MRI images revealed the dose-dependent property of those NPs (Fig. 5a). The transverse relaxivity (r_2_) of FeSe_2_-PEG or BPs-FeSe_2_-PEG was calculated to be 211.65 mM^−1^ s^−1^ or 402.06 mM^−1^ s^−1^, respectively (Fig. 5b). It has been reported that the size, concentration, aggregation and magnetization of contrast agents could affect their performances [63–69]. When the total amount of FeSe_2_ was constant, the clustering of the FeSe_2_ core and the chemical composition of the coating determined enhanced T_2_-weighted imaging ability of BPs-FeSe_2_-PEG. The mPEG-NH_2_ coating of BPs-FeSe_2_-PEG could form hydrogen bonds with water around the material to immobilize water molecules, which affected the nuclear relaxation. The density of FeSe_2_ on BPs was larger than FeSe_2_ alone, which was equivalent to the aggregation of the FeSe_2_ core. The aggregation of magnetic grains could enhance the T_2_ relaxivity of BPs-FeSe_2_-PEG. This indicated that BPs-FeSe_2_-PEG was a promising T_2_ MRI contrast agent. To observe the in vivo MR imaging effect of the nanoparticle more accurately, mice bearing EJ bladder tumors were injected with FeSe_2_-PEG and BPs-FeSe_2_-PEG to monitor the accumulation of NPs in the tumor region. The T_2_-weighted MRI images of tumor areas were obtained. As shown in Fig. 5c, darkening effects were observed in the tumor regions after injection. The effect had been the darkest at 2 h post-injection and then been bright gradually. The intensity of MRI signals in the tumor region showed that the mouse with BPs-FeSe_2_-PEG displayed weaker signal intensity in 24 h than that of FeSe_2_-PEG. The values of T_2_ of BPs-FeSe_2_-PEG in the tumor region were lower than that of FeSe_2_-PEG in Fig. 5d. The MRI signal then gradually increased after 2 h injection, suggesting clearance of the nanosheets from the cancer cells. As a result, the best time point for conducting photothermal therapy was at 2 h post-injection. These results suggested that the BPs-FeSe_2_-PEG could be used to provide precise tumor-specific MRI guidance in tumor theranostics.

### Photothermal therapy of BPs-FeSe_2_-PEG in vivo

**Fig. 6:**
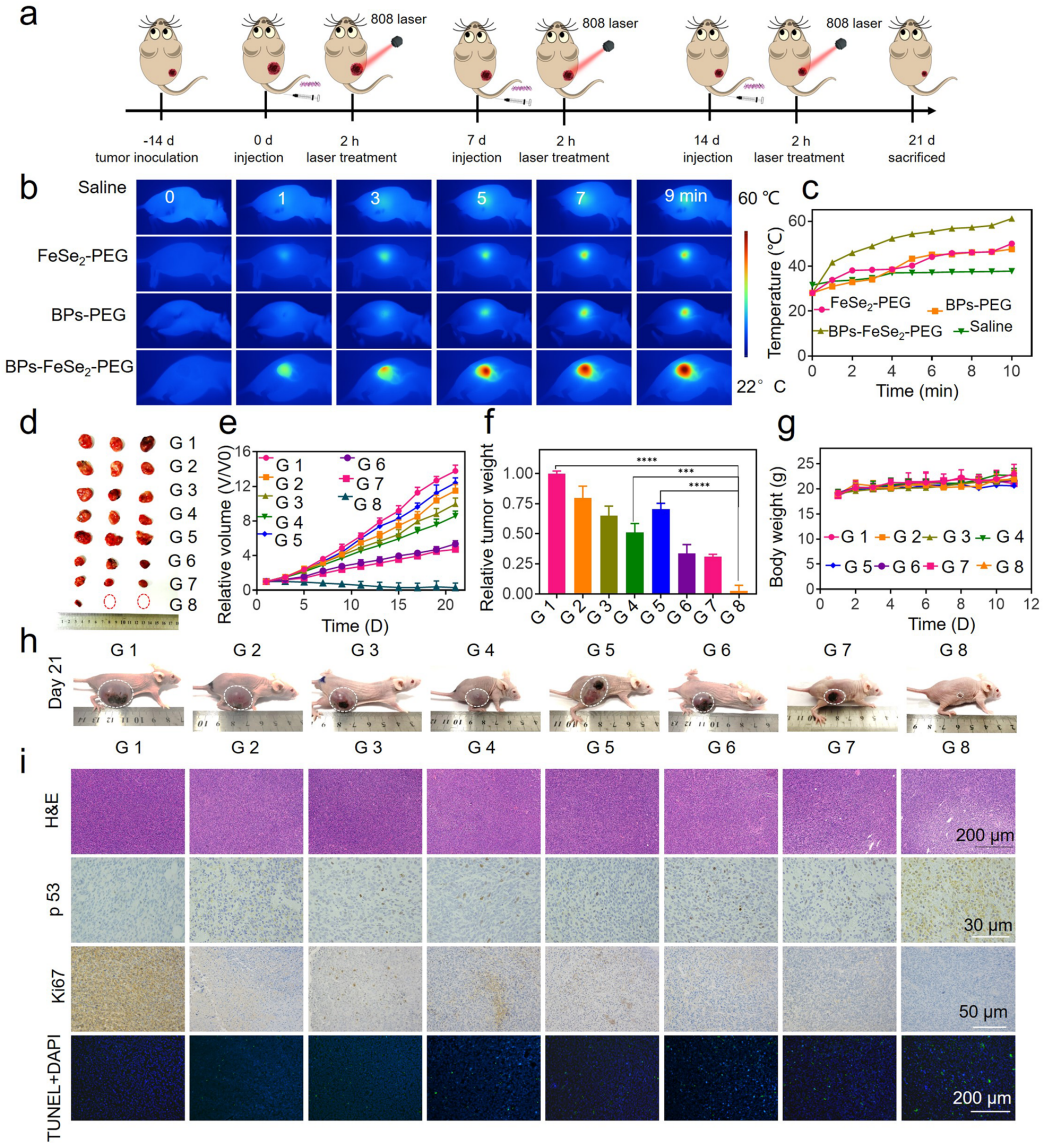
Anticancer efficacy of BPs-FeSe_2_-PEG in vivo. **a** Schematic illustration of BPs-FeSe_2_-PEG (10 mg/kg) under NIR laser (1.5 W/cm^2^, 10 min) to inhabit EJ bladder tumor growth. **b-c** Images and temperature of the tumor region injected with Saline, FeSe_2_-PEG, BPs-PEG and BPs-FeSe_2_-PEG under NIR laser. **d** Photos of tumors after 21 days of treatment. **e** Curves of relative tumor volume during 21 days. **f** Relative tumor weights after 21 days treatment; data are presented as mean ± SD (n = 3), (***P < 0.001, **P < 0.01, *P < *0.05*). **g** The body weight during 21 days treatment. **h** Photos of EJ bladder tumor-bearing mice with/without laser after 21 days. **i** H&E-stained; protein expression levels of p53 and Ki67 in tumor regions of different treatment groups by IHC assay, and TUNEL staining in the tumor region. G 1: saline, G 2: FeSe_2_-PEG; G 3: BPs-PEG; G 4: BPs-FeSe_2_-PEG; G 5: laser; G 6: FeSe_2_-PEG + Laser; G 7: BPs-PEG + Laser; G 8: BPs-FeSe_2_-PEG + Laser

The antitumor efficacy of the BPs-FeSe_2_-PEG in vivo is studied using EJ bladder tumor-bearing nude mice (Fig. 6a). PEGylated FeSe_2_, BPs and BPs-FeSe_2_ nanomaterials were administered via intravenous injection, and NIR laser was irradiated 1.5 W/cm^2^ for 10 min at 2 h post-injection based on the MRI results. Mice were divided randomly into eight groups (n = 3), G 1: saline; G 2: FeSe_2_-PEG; G 3: BPs-PEG; G 4: BPs-FeSe_2_-PEG; G 5: laser; G 6: FeSe_2_-PEG + Laser; G 7: BPs-PEG + Laser; G 8: BPs-FeSe_2_-PEG + Laser. The temperature change of the tumor region with different nanoparticles during the PTT treatment was monitored by a thermal imager. As shown in Fig. 6b, c, the increase of temperature was time-dependent, and the temperature increased to 47.6, 50 and 61.2 °C after injected with FeSe_2_-PEG, BPs-PEG, and BPs-FeSe_2_-PEG, respectively. The tremendous therapeutic effect of BPs-FeSe_2_-PEG upon laser compared with other groups after 21 days of treatment was clearly shown by the images of the tumor region in Fig. 6d. Some of the G 8 group tumors have even been ablated due to the combined effect of BPs-FeSe_2_-PEG and NIR laser. Relative tumor volume and weight changes indicate that the combination of BPs-FeSe_2_-PEG and NIR laser have excellent antitumor effects of inhibiting the growth of EJ bladder tumors (Fig. 6e, f). As shown in Fig. 6g, the bodyweight of all groups shows a stable trend for 21 days, revealing the negligible toxicity of nanoparticles. The changes of tumor volume in each group of mice within 21 days of treatment were recorded in Fig. 6h and Additional file 1: Figure S12. The H&E results of the tumor region further demonstrated that the group treated by the combination of BPs-FeSe_2_-PEG and NIR laser promoted apoptosis of cancer cells in Fig. 6i. Tumor was further analyzed by Immunohistochemistry assay. The p53 plays an important role in inducing apoptosis in cells and regulating the progression of cell cycle [70]. The p53 signaling pathway was remarkably activated in G 8 group indicating the cell apoptosis increased. The G 8 group decreased the expression of Ki67, a marker for proliferation, indicating that tumor cell proliferation was inhibited. Moreover, to further analyze the antitumor efficacy of co-treatment, the apoptosis of tumor sections was detected by TUNEL staining assays. The green fluorescence intensity of G 6, G 7 and G 8 group were relatively higher than other groups, indicating larger numbers of cell apoptosis. The results demonstrated that BPs-FeSe_2_-PEG heteronanostructure had an excellent inhibitory effect on EJ bladder tumors by improving cell apoptosis.

### Evaluation of antitumor effects by MRI

**Fig. 7:**
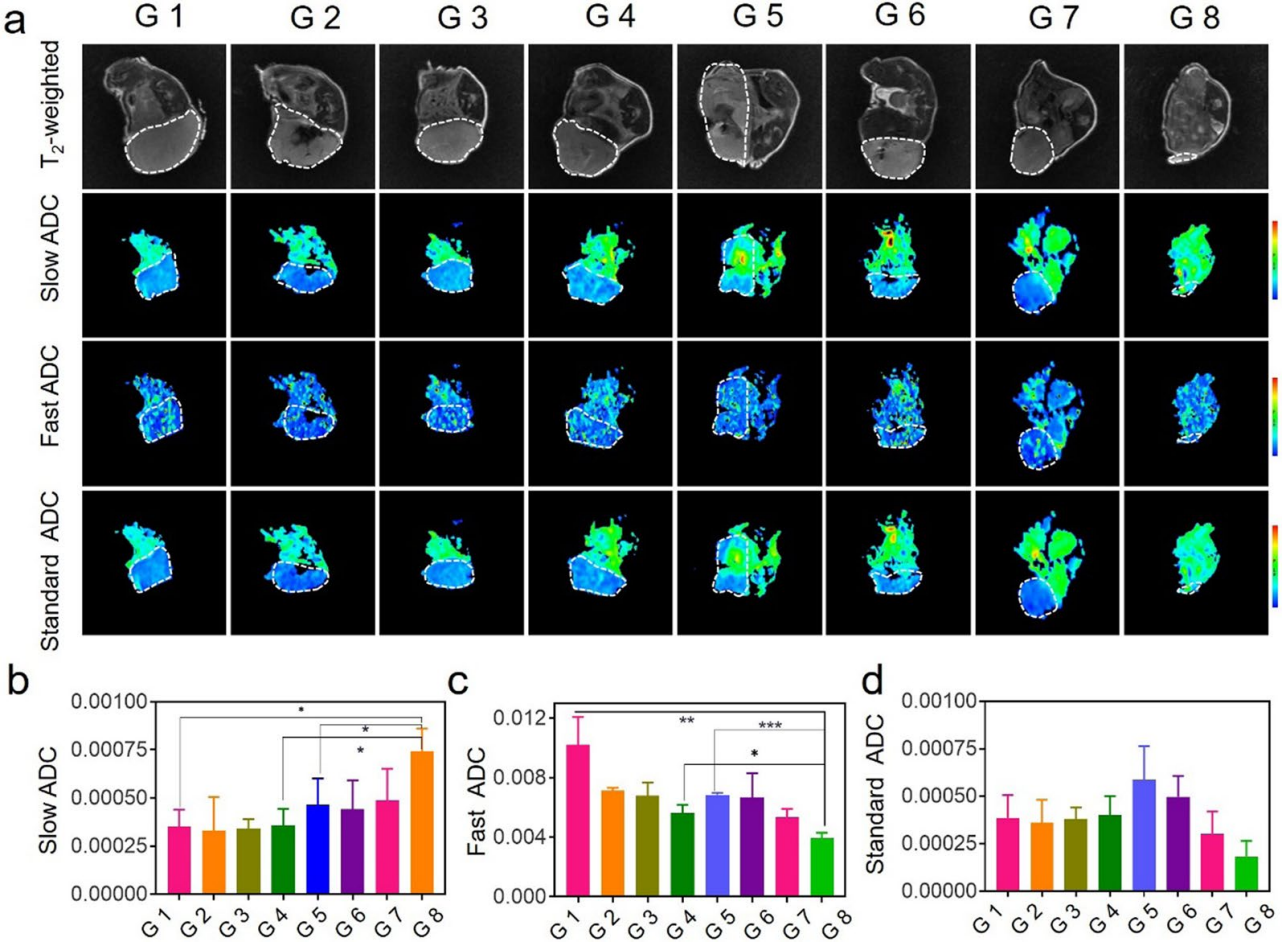
T_2_-weighted imaging analysis of tumor regions of different treatments after 21 days. **a** Images of T_2_-weighted MRI, fast ADC, slow ADC and standard ADC of EJ bladder tumor model with different treatments at 21 days. **b** Values of slow ADC. **c** Value of fast ADC. **d** Value of standard ADC. G 1: saline, G 2: FeSe_2_-PEG; G 3: BPs-PEG; G 4: BPs-FeSe_2_-PEG; G 5: laser; G 6: FeSe_2_-PEG + Laser; G 7: BPs-PEG + Laser; G 8: BPs-FeSe_2_-PEG + Laser. Data are presented as means ± SD (n = 3), (***P < 0.001, **P < 0.01, or *P < *0.05)*

The antitumor activity of BPs-FeSe_2_-PEG was further evaluated by MRI. We obtained T_2_-weighted MRI images of tumor areas of mice in different treatment groups after 21 days. BPs-FeSe_2_-PEG cotreatment group presented the smallest tumor area than that of other groups after 21 days shown in Fig. 7a. Besides, the cell density and activity of tumor regions could be evaluated by slow ADC value. As shown in Fig. 7b, compared with the other groups, the enhanced slow ADC values in the tumor region of G 8 indicated a decrease in the density and activity of bladder tumor cells. Moreover, fast ADC signals are related to the blood flow in the tumor area in Fig. 7c. A weaker fast ADC intensity can be observed at the tumor region in the BPs-FeSe_2_-PEG combined with the NIR laser treatment group. As shown in Fig. 7d, the value of standard ADC in G 8 decreases, demonstrating the excellent anticancer activity of BPs-FeSe_2_-PEG. In summary, BPs-FeSe_2_-PEG heteronanostructure combined with laser could significantly inhibit EJ bladder tumor growth in vivo.

### Toxicity evaluation of BPs-FeSe_2_-PEG in vivo

**Fig. 8:**
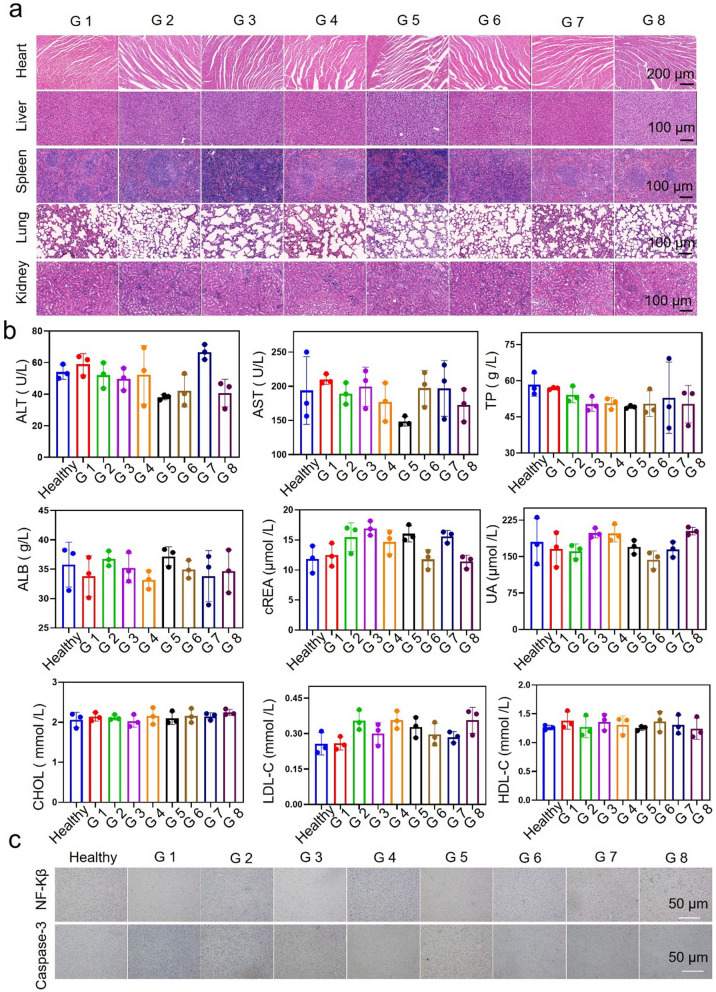
Toxicity evaluation of BPs-FeSe_2_-PEG in vivo. **a** H&E staining of main organs of the mice under different treatments for 21 days. **b** ALT; AST; TP; ALB; cREA; UA; CHOL; LDL-C and HDL-C in all groups of mice with different treatments for 21 days (n = 3). **c** Hepatic NF-kβ expression and caspase-3 expression of different groups of mice by IHC assay. G 1: saline, G 2: FeSe_2_-PEG; G 3: BPs-PEG; G 4: BPs-FeSe_2_-PEG; G 5: laser; G 6: FeSe_2_-PEG + Laser; G 7: BPs-PEG + Laser; G 8: BPs-FeSe_2_-PEG + Laser

It is crucial to evaluate the safety of BPs-FeSe_2_-PEG in vivo. All of the mice were sacrificed to collect their main organs and blood after 21 days of treatment. As shown in Fig. 8a, the main organs of H&E-stained results of the combined BPs-FeSe_2_-PEG and laser exhibited no detectable toxicity to the major organs. Besides, as shown in Fig. 8b and Additional file 1: Figure S13, the biochemical indicator level of G 8 group of alanine aminotransferase (ALT), aspartate aminotransferase (AST), total protein (TP), globulin (GLOB), cholesterol (CHOL), uric acid (UA), Creatinine (CREA), urea (UREA), Glucose (GLU), high-density lipoprotein cholesterol (HDL-C), low-density lipoprotein cholesterol (LDL-C), triglyceride (TG), creatine kinase (CK), Lactate dehydrogenase (LDH) and albumin (ALB) showed no obviously difference compared with that of the healthy group.

The biodistribution of BPs-FeSe_2_-PEG in the main organs and tumors of EJ bladder tumor mouse model was evaluated to investigate the longer term biodistribution. The concentration of Se element after 21 days of treatment was measured by ICP-MS. As shown in Additional file 1: Figure S14, BPs-FeSe_2_-PEG could accumulate in liver and tumor areas, suggesting that the liver was the main target organ and the BPs-FeSe_2_-PEG could accumulate in tumor region for MRI-guided PTT. Besides, the results demonstrated that BPs-FeSe_2_-PEG could be degraded and excreted over time and the BPs-FeSe_2_-PEG was safe and had no accumulative toxicity. Besides, the pharmacokinetics of BPs-FeSe_2_-PEG in vivo was studied. Then we used ICP-MS to measure the Se concentration in the blood of mice injected with 4 mg/kg BPs-FeSe_2_-PEG [71]. The blood was obtained at 0.5, 1, 2, 4, 8, 12, 24, 48 and 72 h after injection. As shown in Additional file 1: Figure S15 and Table S1, the plasma Se concentration in blood sharply decreased and decayed to half in 17 h, suggesting that the nanoparticle was degraded and excreted over time. These results exhibit that the BPs-FeSe_2_-PEG has good degradability in vivo*.*

The liver is the metabolic organ. Nanoparticles could penetrate the cell membrane into cells, through lipid peroxidation or promote the production of reactive oxygen species and other ways to cause liver injury, affecting the normal physiological function of the liver. The effects of BPs-FeSe_2_-PEG heterodimer on liver inflammation and apoptosis in mice were evaluated by analyzing the expression of NF-Kβ and caspase-3. The NF-kβ receptor is a cytoplasmic protein complex responsible for regulating the expression of a variety of inflammatory mediators. Caspase-3 is considered as the central regulator of apoptosis, and the activated caspase-3 signal pathway induces apoptosis of tumor cells. It should be noted that the expression of NF-Kβ and caspase-3 in the liver of all groups of mice was negative in Fig. 8c. These results indicate the BPs-FeSe_2_-PEG heteronanostructure does not cause liver inflammation and apoptosis and has no obvious hepatotoxicity. In summary, BPs-FeSe_2_-PEG is an effective and safe nanoparticle to inhibit the growth of EJ bladder tumors.

## Conclusions

In summary, we have designed a BP-based heteronanostructure nanoparticle system and demonstrated its enhanced photothermal conversion efficiency mechanism, good photostability, MRI property and excellent antitumor effects. The mechanism reveals that BPs-FeSe_2_-PEG heteronanostructure could enhance separation of photoinduced electron–hole pairs, accelerate charge transfer and reduce the recombination rate of photoinduced carriers, resulting in the generation of phonons to release excess energy to return to equilibrium state via non-radiative recombination at the interface of FeSe_2_ and BPs, thus leading to higher photothermal conversion than free FeSe_2_ and BPs. The presence of Fe enables BPs-FeSe_2_-PEG to be used as an MRI agent for imaging-directed photothermal treatment. The aggregation of FeSe_2_ on the surface of BPs and the formation of hydrogen bonds could enhance the T_2_ relaxivity of BPs-FeSe_2_-PEG. Besides, MRI in tumor help to choose the best time point for photothermal therapy. Finally, BPs-FeSe_2_-PEG heteronanostructure shows remarkable photoablation of tumors and has no obvious toxicity to major organs. In summary, this work provides a novel strategy for fabricating BPs-based heteronanostructure nanomaterials that can simultaneously enhance photothermal conversion efficiency and photostability and realize MRI for precise cancer therapeutic efficacy. Our strategy paves a new path to design black phosphorus-based heterostructures for biomedical applications, integrated diagnosis and therapy.

## Supplementary Information

**Additional file 1: Figure S1.** AFM and TEM images of BPs and BPs-FeSe_2_ with different thicknesses. **Figure S2.** TEM images of BPs-FeSe_2_ nanostructures prepared with various FeSe_2_: BPs feeding ratios. **Figure S3.** UV–vis absorption spectra of BPs-FeSe_2_. **Figure S4.** Photographs of (a) FeSe_2_, BPs and BPs-FeSe_2_; (b) PEGylated FeSe_2_, BPs and BPs-FeSe_2._ **Figure S5.** The UV–vis absorption spectra of PEGylated FeSe_2_, BPs and BPs-FeSe_2_ solutions during 14 days. **Figure S6.** TEM images of BPs-FeSe_2_-PEG under different pH conditions. **Figure S7.** Temperature curves of FeSe_2_-PEG and BPs-PEG**. Figure S8.** Infrared thermal images and temperature change of BPs-FeSe_2_-PEG in the 96-hole plate. **Figure S9.** The SVHUC-1 cell viability of PEGylated FeSe_2_, BPs and BPs-FeSe_2_ at various concentrations. **Figure S10.** Fluorescence images of EJ cells treated with coumarin-6-loaded BPs-FeSe_2_-PEG. **Figure S11.** The changes of H_2_O_2_ level in EJ bladder cells under different treatment **Figure S12.** Photos of EJ bladder tumor-bearing mice with/without laser at different time points. **Figure S13.** Hematological analysis of mice. **Figure S14.** The in vivo biodistribution of Se concentration. **Figure S15.** Plasma Se concentration. **Table S1** Pharmacokinetic parameters of BP-FeSe_2_-PEG.

## Data Availability

All data generated or analyzed during this study are included in this article.

## Abbreviations

FeSe_2_: Ferrous selenide
BPs: Black phosphorus nanosheets
mPEG-NH_2_: Methoxy poly (ethylene glycol)
XRD: X-ray powder diffraction
XPS: X-ray photoelectron spectroscopy
TEM: Transmission electron microscopy
HR-TEM: High-resolution TEM
AFM: Atomic force microscope
EDS: Energy-dispersive X-ray spectroscopy
η: Photothermal conversion efficiency
MTT: 3-(4, 5-Dimethylthiazol-2-yl)-2, 5-diphenyltetrazolium bromide
DCFH-DA: 2, 7-Dichlorodihydrofluorescein diacetate
DPBF: 1, 3-Diphenylisobenzofuran
ROS: Reactive oxygen species
^−^O_2_: Superoxide anions
.OH: Hydroxyl radical
H_2_O_2_: Hydrogen peroxides
^1^O_2_: Singlet oxygen
r_2_: Transverse relaxivity
ALT: Alanine aminotransferase
AST: Aspartate aminotransferase
TP: Total protein
GLOB: Globulin
CHOL: Cholesterol
UA: Uric acid
CREA: Creatinine
UREA: Urea
GLU: Glucose
HDL-C: High-density lipoprotein cholesterol
LDL-C: Low-density lipoprotein cholesterol
TG: Triglyceride
CK: Creatine kinase
LDH: Lactate dehydrogenase
ALB: Albumin
G 1: Saline
G 2: FeSe_2_-PEG
G 3: BPs-PEG
G 4: BPs-FeSe_2_-PEG
G 5: Laser
G 6: FeSe_2_-PEG + Laser
G 7: BPs-PEG + Laser
G 8: BPs-FeSe_2_**-**PEG + Laser
ICP-MS: Inductively coupled plasma mass spectrometry
