# Supplementary material for: Designing highly stable ferrous selenide-black phosphorus nanosheets heteronanostructure via P-Se bond for MRI-guided photothermal therapy
Source: J Nanobiotechnology. 2021 Jul 6;19:201. doi: 10.1186/s12951-021-00905-5 (PMC8262019; doi:10.1186/s12951-021-00905-5)
Supplement: Supplementary file 1 — Additional file 1: Figure S1. AFM and TEM images of BPs and BPs-FeSe2 with different thicknesses. Figure S2. TEM images of BPs-FeSe2 nanostructures prepared with various FeSe2: BPs feeding ratios. Figure S3. UV–vis absorption spectra of BPs-FeSe2. Figure S4. Photographs of (a) FeSe2, BPs and BPs-FeSe2; (b) PEGylated FeSe2, BPs and BPs-FeSe2. Figure S5. The UV–vis absorption spectra of PEGylated FeSe2, BPs and BPs-FeSe2 solutions during 14 days. Figure S6. TEM images of BPs-FeSe2-PEG under different pH conditions. Figure S7. Temperature curves of FeSe2-PEG and BPs-PEG. Figure S8. Infrared thermal images and temperature change of BPs-FeSe2-PEG in the 96-hole plate. Figure S9. The SVHUC-1 cell viability of PEGylated FeSe2, BPs and BPs-FeSe2 at various concentrations. Figure S10. Fluorescence images of EJ cells treated with coumarin-6-loaded BPs-FeSe2-PEG. Figure S11. The changes of H2O2 level in EJ bladder cells under different treatment Figure S12. Photos of EJ bladder tumor-bearing mice with/without laser at different time points. Figure S13. Hematological analysis of mice. Figure S14. The in vivo biodistribution of Se concentration. Figure S15. Plasma Se concentration. Table S1 Pharmacokinetic parameters of BP-FeSe2-PEG. [file 12951_2021_905_MOESM1_ESM.docx]

Designing Highly Stable Ferrous Selenide-Black Phosphorus Nanosheets Heteronanostructure *via* P-Se Bond for MRI Imaging-Guided Photothermal Therapy

Xuanru Deng ^#,^ ^1^, Hongxing Liu ^#, 1, 2, 3^ Yuan Xu ^2^, Leung Chan ^1, 2^, Jun Xie ^1^, Zushuang Xiong ^1^, Zheng Tang ^1^, Fang Yang* ^1^, Tianfeng Chen* ^1, 2^

*^1^ College of Chemistry and Materials Science, Guangdong Provincial Key Laboratory of Functional Supramolecular Coordination Materials and Applications, Jinan University, Guangzhou 510632, Jinan University; China*

*^2^ Medical Imaging Center, The First Affiliated Hospital, Jinan University, Guangzhou 510632, Jinan University; China*

*^3^ Department of Urology, Guangzhou Institute of Urology, Guangdong Key Laboratory of Urology, the First Affiliated Hospital of Guangzhou Medical University*

*Guangzhou Medical University, Guangzhou 510230, China.*

* Corresponding author.

Email: [tchentf@jnu.edu.cn](mailto:tchentf@jnu.edu.cn), [tyoung@jnu.edu.cn](mailto:tyoung@jnu.edu.cn).

^#^ Equal contribution

**Additional file 1:**


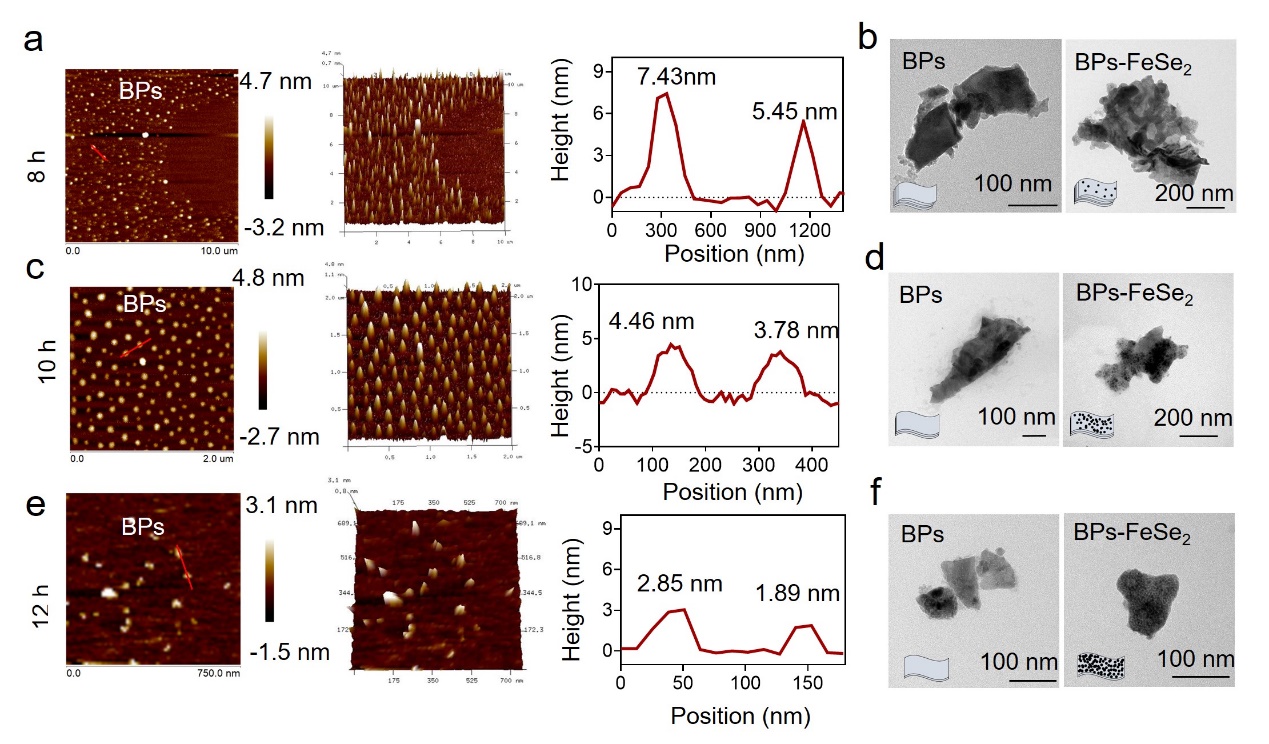


**Figure S1**. (**a**) 2 D and 3 D AFM images of BPs ultrasound for 8 h and the AFM-measured height profile of BPs showed a thickness of about 8.5 nm. (**b**) TEM images of BPs and BPs-FeSe_2_.

(**c**): 2 D and 3 D AFM images of BPs ultrasound for 10 h and the AFM-measured height profile of BPs, which showed a thickness of about 4.4 nm. (**d**) TEM images of BPs and BPs-FeSe_2_.

(**e**): 2 D and 3 D AFM images of BPs ultrasound for 12 h and the AFM-measured height profile of BPs, which showed a thickness of about 2.5 nm. (**f**) TEM images of BPs and BPs-FeSe_2_.


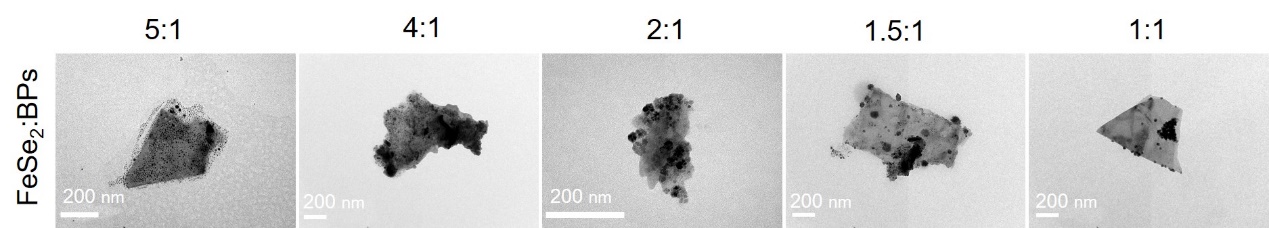


**Figure S2.** TEM images of BPs-FeSe_2_ nanostructures prepared with various FeSe_2_: BPs feeding ratios.


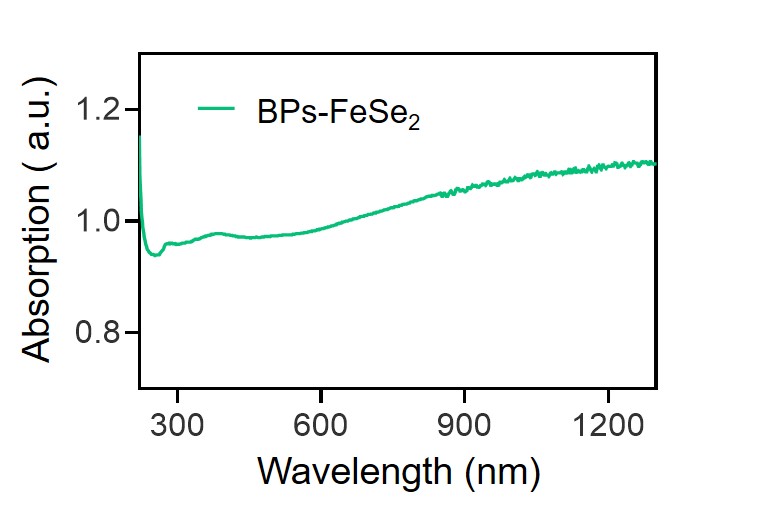


**Figure S3.** UV-vis absorption spectra of BPs-FeSe_2_.


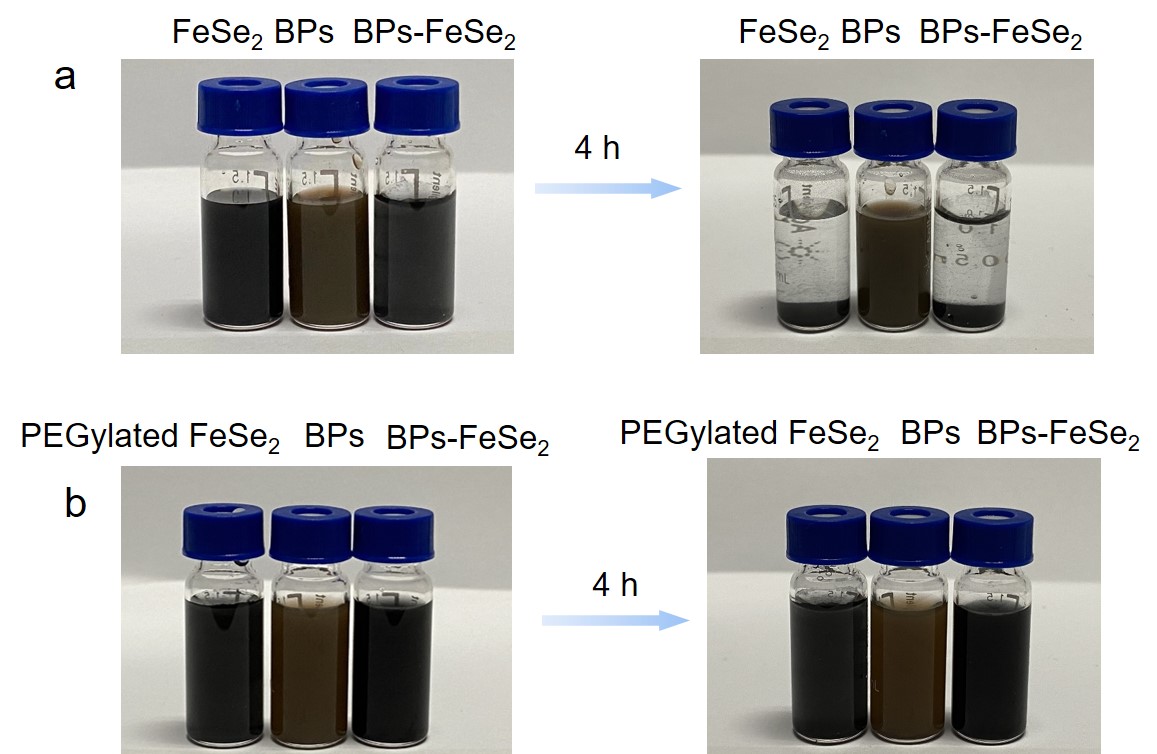


**Figure S4**. Photographs of (**a**) FeSe_2_, BPs and BPs-FeSe_2_; (**b**) PEGylated FeSe_2_, BPs and BPs-FeSe_2._


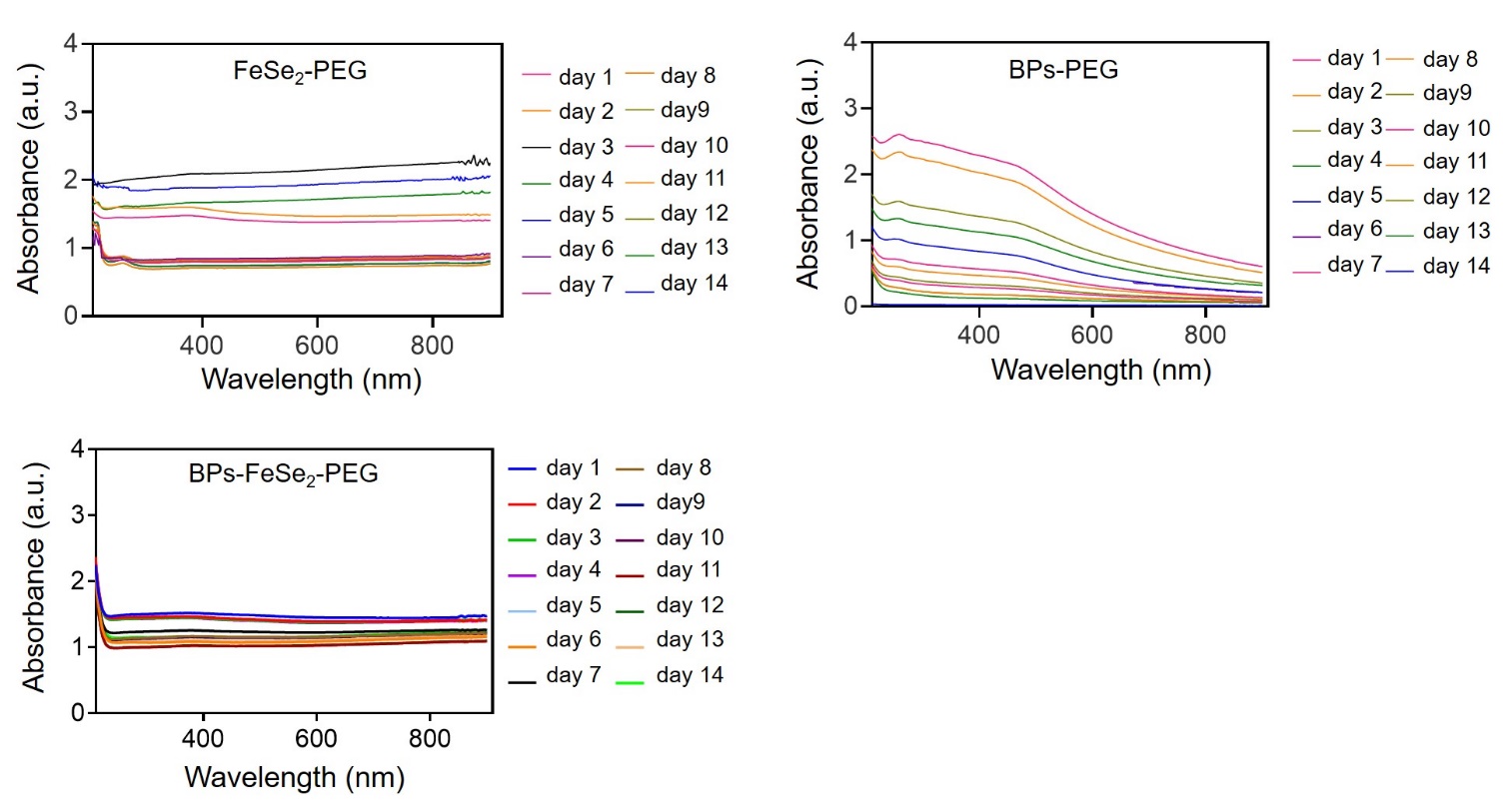


**Figure S5**. UV-vis absorption spectra of PEGylated FeSe_2_, BPs and BPs-FeSe_2_ solutions during 14 days.


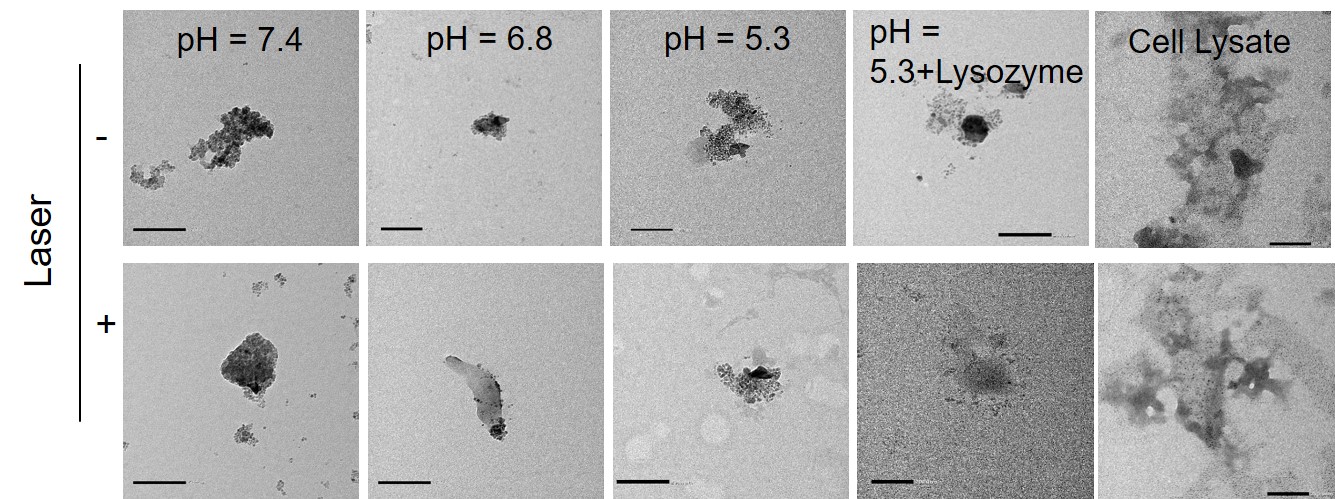


**Figure S6**. TEM images of BPs-FeSe_2_-PEG (1 mg/mL, 1 mL) in different buffers for 24 h.


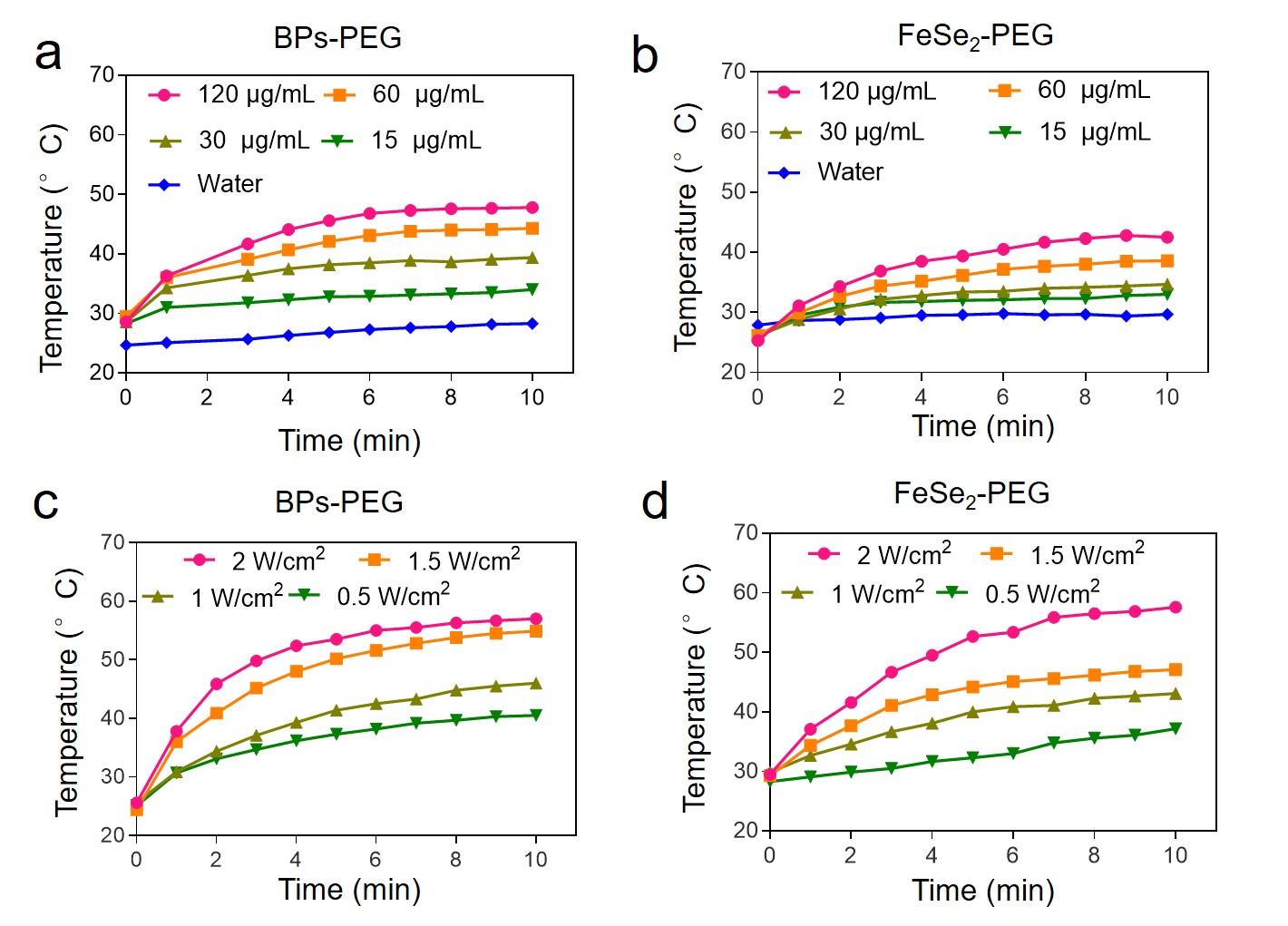


**Figure S7**. (**a**) Temperature curves of BPs-PEG (120, 60, 30, 15 μg/mL) under the irradiation (1.5 W/cm^2^, 10 min); (**b**) Temperature curves of FeSe_2_-PEG (120, 60, 30, 15 μg/mL) under the irradiation (1.5 W/cm^2^, 10 min). (**c**) Temperature curves of BPs-PEG (120 μg/mL) under the irradiation of NIR laser (0.5, 1, 1.5, 2 W/cm^2^, 10 min); (**d**) Temperature curves of FeSe_2_-PEG (120 μg/mL) under the irradiation of NIR laser (0.5, 1, 1.5, 2 W/cm^2^, 10 min).


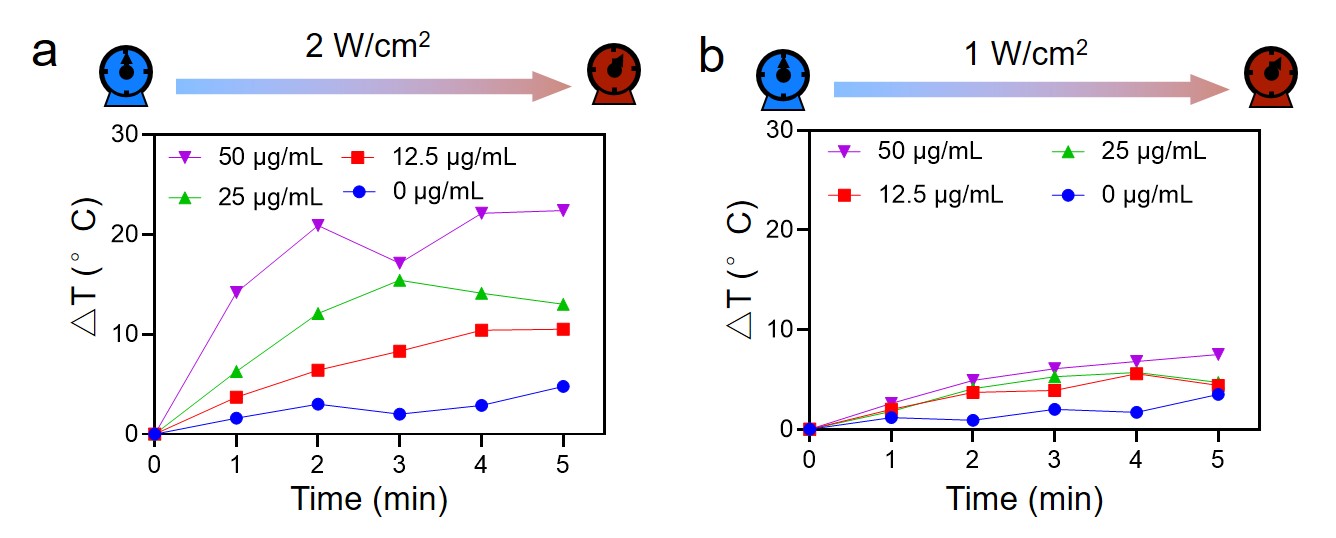


**Figure S8.** (**a, b**) Temperature change of EJ cells incubated with different concentrations (50, 25, 12.5 and 0 μg/mL) BPs-FeSe_2_-PEG in the 96-well hole under NIR laser (2 W/cm^2^, 5 min, and 1 W/cm^2^, 5 min).


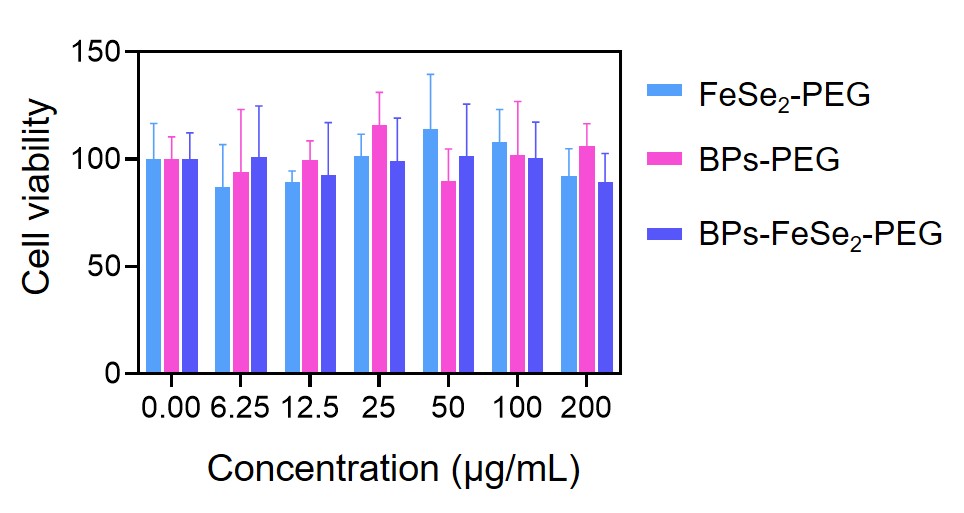


**Figure S9**. The SVHUC-1 cell viability of PEGylated FeSe_2_, BPs and BPs-FeSe_2_ without laser for 72 h.


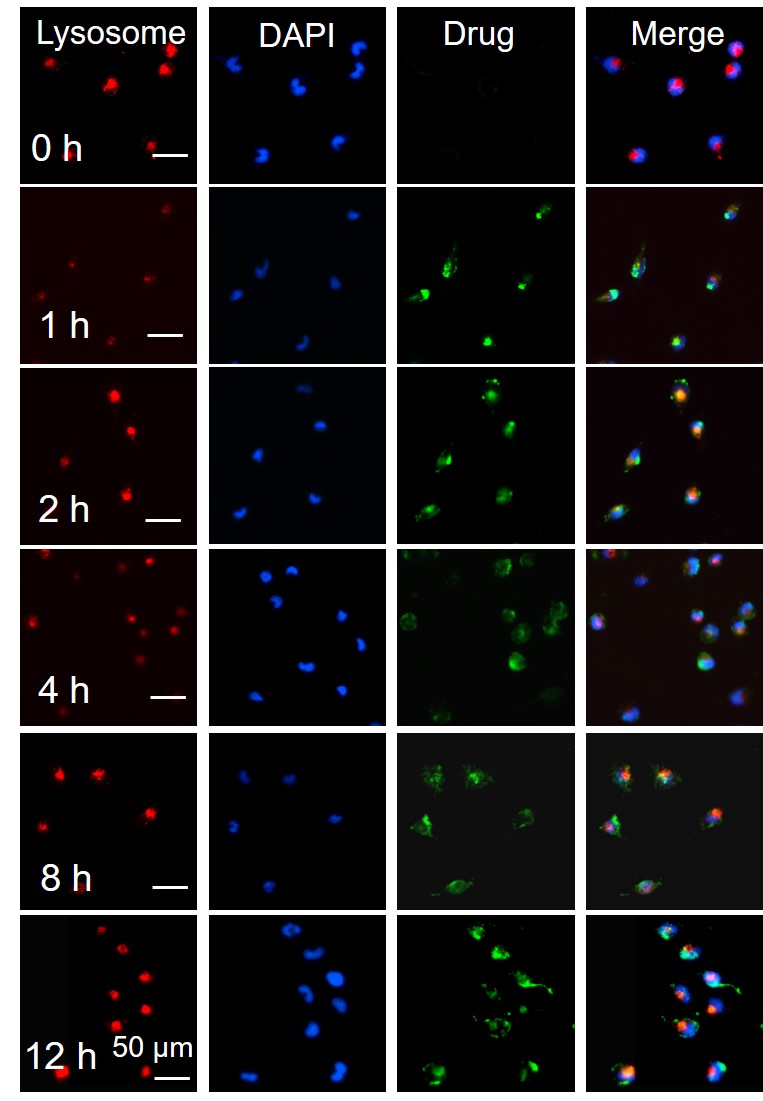


**Figure S10**. Fluorescence images of EJ cells treated with coumarin-6-loaded BPs-FeSe_2_-PEG (25 μg/mL) and stained with lysotracker (lysosome) and DAPI (nucleus) for 0, 1, 2, 4, 8 and 12 h, respectively.


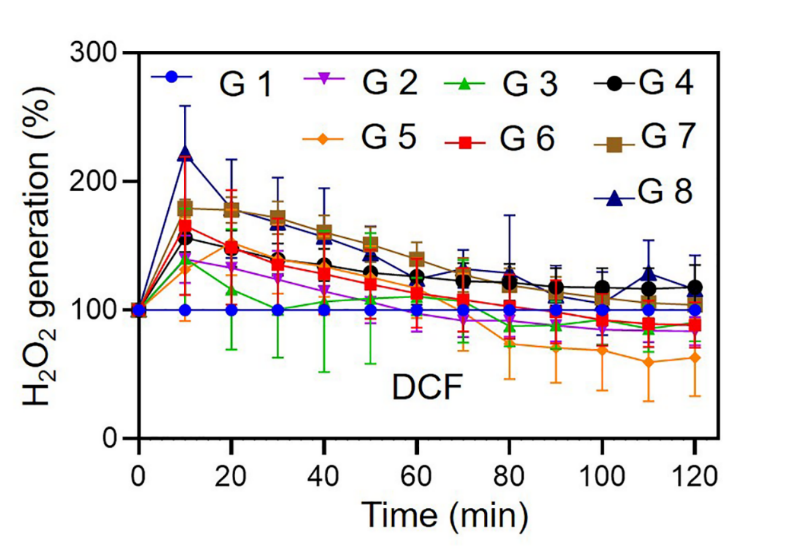


**Figure S11**. The changes of H_2_O_2_ level in EJ bladder cells during 120 min under different treatment. G 1: Control, G 2: FeSe_2_-PEG; G 3: BPs-PEG; G 4: BPs-FeSe_2_-PEG; G 5: Laser; G 6: FeSe_2_-PEG + Laser; G 7: BPs-PEG + Laser; G 8: BPs-FeSe_2_-PEG +Laser (100 μg/mL, 1.5 W/cm^2^, 1 min).


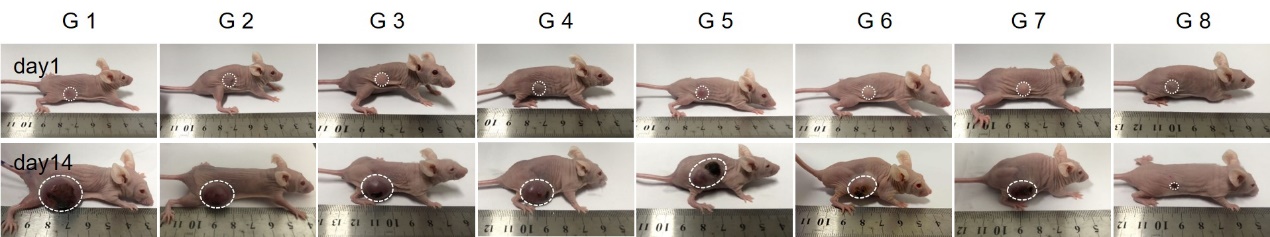


**Figure S12**. Photos of EJ bladder tumor-bearing mice with/without laser at different time points (1.5 W/cm^2^, 10 min). G 1: saline, G 2: FeSe_2_-PEG; G 3: BPs-PEG; G 4: BPs-FeSe_2_-PEG; G 5: Laser; G 6: FeSe_2_-PEG + Laser; G 7: BPs-PEG + Laser; G 8: BPs-FeSe_2_-PEG +Laser (10 mg/kg).


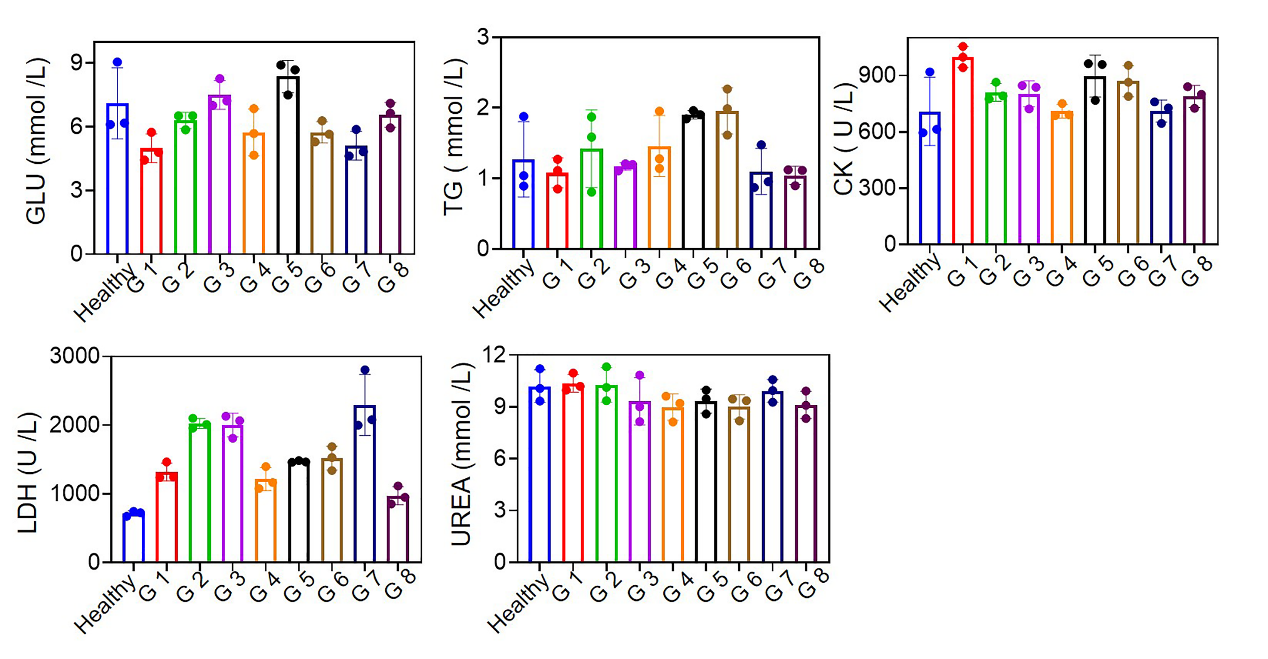


**Figure S13**. The change in GLU, TG, CK, LDH and UREA in all groups of mice with different treatments for 21 days (n=3).


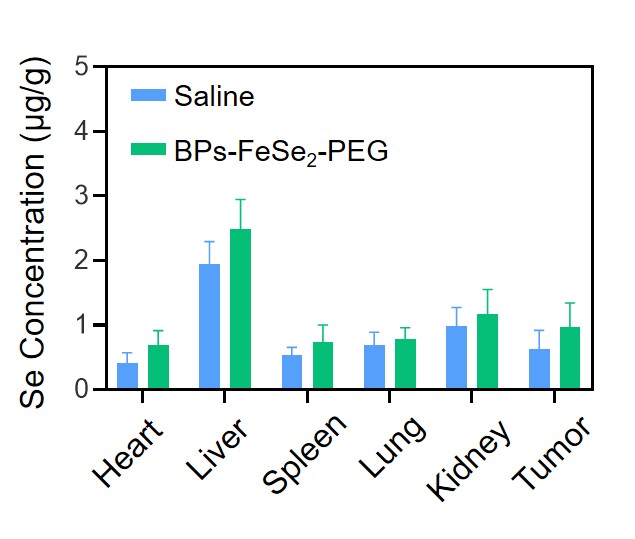


**Figure S14.** The *in vivo* biodistribution of Se concentration of BPs-FeSe_2_-PEG (10 mg/kg) in different organs after 21 days of treatment (n = 3).


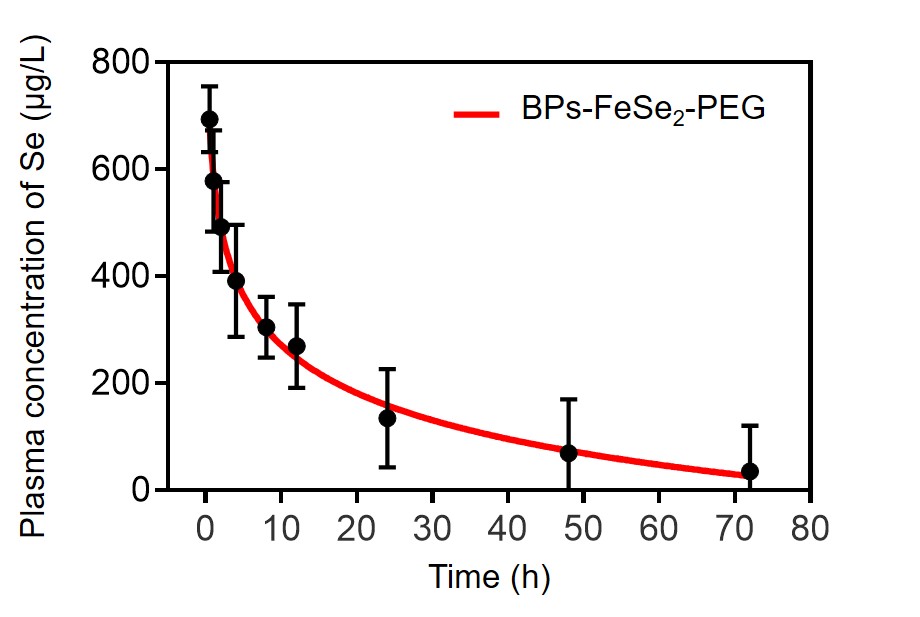


**Figure S15.** Plasma Se concentration versus time after intravenous injection (0.5, 1, 2, 4, 8, 12, 24, 48 and 72 h) of BPs-FeSe_2_-PEG (4 mg/kg).

**Table S1** Pharmacokinetic parameters of BPs-FeSe_2_-PEG (4 mg/kg) after intravenous injection.


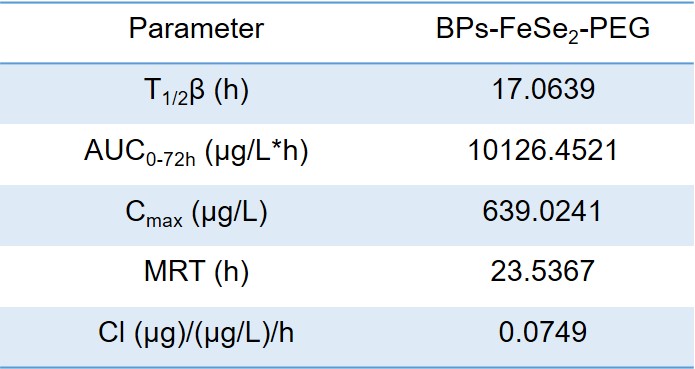


T_1/2_β, elimination phase, half-life period of medicine

AUC_0-72 h_, area under the concentration versus time curve.

C_max_, maximum concentration observed.

MRT, mean retention time.

Cl, clearance of medicine.
